# A masking clamp for conditional activation of therapeutic antibodies

**DOI:** 10.3389/fimmu.2025.1640427

**Published:** 2025-10-30

**Authors:** Adrian Bloch, Jan Felix Zimmermann, Jan Habermann, Evelyn Ullrich, Harald Kolmar

**Affiliations:** ^1^ Institute for Organic Chemistry and Biochemistry, Technical University of Darmstadt, Darmstadt, Germany; ^2^ Goethe University, Frankfurt am Main, Germany; ^3^ Frankfurt Cancer Institute (FCI), Goethe University, Frankfurt am Main, Germany; ^4^ German Cancer Consortium (DKTK), Partner Site Frankfurt/Mainz and German Cancer Research Center (DKFZ), Heidelberg, Germany; ^5^ Mildred Scheel Career Center (MSNZ), Hospital of the Goethe University Frankfurt, Frankfurt am Main, Germany; ^6^ Centre for Synthetic Biology, Technical University of Darmstadt, Darmstadt, Hesse, Germany

**Keywords:** antibody engineering, antibody masking, conditional antibody activation, off-target toxicity, MMP-9

## Abstract

Therapeutic monoclonal antibodies (mAbs) constitute cornerstone therapeutics in oncology, yet their clinical utility is often limited by on-target, off-tumor toxicity due to shared antigen expression in both tumor and healthy tissues. To counteract this issue, various approaches, including pH-dependent, as well as affinity-based and steric hindrance-based masked antibodies, have been developed. Several steric hindrance-based masking strategies have been proposed utilizing non-human proteins, potentially leading to an immunogenic response. To address this challenge, we engineered a modular protein-based masking platform leveraging the high-affinity interaction between human calmodulin (CaM) and a calmodulin-binding peptide (CBP). This strategy enables conditional activation of antibodies *via* tumor microenvironment (TME)-associated proteases (e.g., MMP-9), minimizing systemic off-tumor binding. The CaM-CBP peptide clamp, composed exclusively of human-derived protein domains, was fused to the amino termini of heavy and light chains of trastuzumab and cetuximab. On-cell binding assays demonstrated up to a 410-fold reduction in EC_50_ for masked constructs across multiple antigen-antibody systems. Functional validation using a reporter-cell-based antibody-dependent cellular cytotoxicity (ADCC) assay confirmed that masking abrogated effector cell activation, leading to up to 78-fold reduction of EC_50_ and no ADCC activation at concentrations corresponding to the onset of maximal ADCC activation by unmodified antibodies. Demasking *via* MMP-9-mediated linker hydrolysis restored antigen binding and ADCC potency. Structural optimization revealed that linker length and clamp positioning critically influenced masking efficiency. This human-derived, modular masking platform mitigates immunogenicity risks while enabling tumor-selective antibody activation. Its adaptability across antibody scaffolds underscores broad applicability for improving the therapeutic index of antibodies.

## 1 Introduction

Cancer therapy has undergone a profound transformation in recent decades, evolving from surgery and radiotherapy to the advent of chemotherapy and, more recently, to highly targeted biological agents such as monoclonal antibodies (mAbs) and personalized immunotherapies including chimeric antigen receptor (CAR) T cell therapies (1–3). mAbs, in particular, have revolutionized oncology by offering the potential for selective tumor targeting while minimizing collateral damage to healthy tissues. However, this specificity is not absolute; tumor-associated antigens (TAAs) recognized by therapeutic mAbs are often also expressed on normal cells, leading to on-target, off-tumor toxicity that can be dose-limiting and compromise therapeutic efficacy (4–6). To address this challenge, innovative strategies have been developed to mask antibody binding domains and enable conditional activation within the TME (7, 8). These approaches typically involve the attachment of a masking domain *via* a flexible linker to the N-terminus of the antibody heavy or light chain, thereby sterically blocking antigen engagement in healthy tissues. Upon exposure to TME-specific stimuli, such as elevated protease activity or altered pH, the mask is removed or rendered non-functional, restoring antibody binding and effector functions (7, 9–11). Current masking strategies can be broadly categorized into two mechanistic classes: steric hindrance-based and affinity-based masks. Affinity-based masks employ domains such as anti-idiotypic single-chain variable fragments (scFvs), nanobodies^®^, or affinity peptides, which bind specifically to the antibody paratope and require custom engineering for each target (9, 12–15). In contrast, steric hindrance-based masks function independently of specific antibody-antigen interactions, offering modularity and facilitating the transfer of masking domains between different antibody scaffolds. This modularity streamlines the development of masked antibody therapeutics and expands their applicability across diverse oncology targets.

We recently masked the therapeutic antibody 6G11 (BI-1206) by isolating an anti-idiotypic scFv from a chicken immunization library and fusing the molecule to the N-terminus of the V_L_. This modification effectively blocked the interaction between 6G11 and Fcγ receptor IIB. However, the affinity of the masking scFv required rational engineering to ensure that antigen binding could be fully restored following protease-mediated demasking, specifically *via* MMP-9 cleavage (15). A prominent example of an affinity peptide-based approach is the cetuximab probody PB1 by CytomX. By fusing a blocking peptide identified from a bacterial peptide display library to the amino terminus of V_L_ of cetuximab, binding to EGFR was diminished and could be restored upon incubation with proteases (10). This affinity peptide-based masking approach was then used to generate a conditional PD-1/PD-L1 probody and a masked anti-CD166 ADC (16). In Phase I/II trials, the masked ADC demonstrated translational and clinical activity in a variety of different tumor types (17). While effective, such affinity-based masks are always antibody-specific and require custom optimization for each target.

In contrast, steric hindrance-based masking approaches offer broader applicability. For instance, Seagen engineered a generic masking platform by fusing heterodimeric coiled-coil domains to the amino termini of the heavy and light chains of therapeutic antibodies, separated by MMP-2/-9-cleavable linkers. Masking HER2 and CD-19 targeting antibodies with a WinZip-A1B1-derived coiled-coil from a library approach resulted in a 753-fold reduction in on-cell EC_50_ for HER2-positive SK-BR-3 cells and led to diminished internalization and complement-dependent cellular cytotoxicity (CDC) in CD19-positive Raji cells (18). This masking approach was then transferred to an anti-CD47 antibody, generating the masked antibody SGN-CD47M. Based on promising results in tolerability studies in cynomolgus monkeys and anti-tumor activity in xenograft mouse models, this therapeutic has recently advanced into phase I dose-escalation clinical trials (19). Beyond protease activation, the unique biochemical features of the TME, such as acidic pH, can also be leveraged for conditional antibody activation. For example, engineering a pH-sensitive variant of the *Mycoplasma genitalium*-derived protein M enabled the reversible masking of trastuzumab. The engineered protein M binds the Fv light chain domain with high affinity at physiological pH, blocking antigen binding, but dissociates at lower pH, partially restoring binding to HER2-positive cells in a time-dependent manner (11).

**Figure 1:**
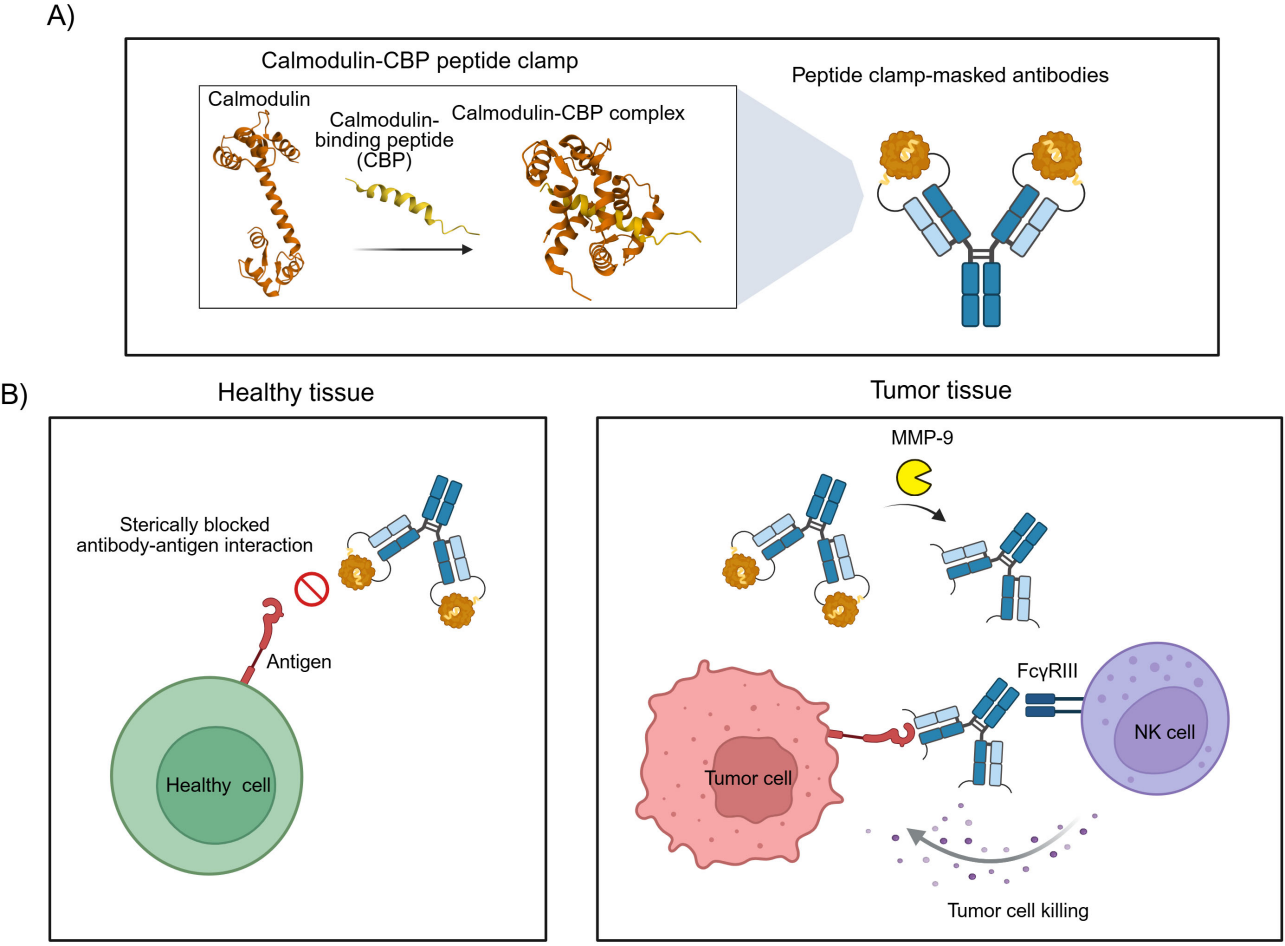
Design and mode of action of antibodies masked with a peptide clamp. **(A)** Scheme for the masking of therapeutic antibodies with a peptide clamp to generate conditionally active antibodies. By fusing a peptide clamp consisting of calmodulin and calmodulin-binding peptide (CBP) to the N-terminal domain of the heavy and light chain of an antibody *via* an MMP-9 cleavable linker, masked antibodies are generated. Calmodulin binds to calmodulin-binding peptide, forming a globular CaM-CBP complex. **(B)** Conceptual illustration of the mode of action of peptide clamp-masked antibodies. In healthy tissue, the binding of the antibody to its antigen is prevented due to steric hindrance in the antibody-antigen interaction introduced by the peptide clamp. Thereby, on-target, but off-tumor side effects are reduced (left panel). Entering the tumor microenvironment, matrix metalloproteinases cleave off the peptide clamp. Removal of the mask restores antibody binding and can enable FcγRIII-mediated killing of tumor cells by natural killer (NK) cells (right panel). Structures used in Figure A: Calmodulin (PDB: 1CLL) and CaM-CBP complex (PDB: 2LV6). Created with Biorender.com.

Steric hindrance-based masking strategies for therapeutic antibodies offer distinct advantages over affinity-based masking, including broad modularity, accelerated development timelines, and simplified manufacturing processes. However, current implementations of such approaches often employ masking domains of non-human origin, which could elicit undesirable immunogenic responses in patients. To address this limitation, we sought to develop a generic masking platform using exclusively human-derived proteins. Our strategy exploits the high-affinity interaction between calmodulin (CaM) and a calmodulin-binding peptide (CBP) derived from the carboxy-terminal domain of skeletal muscle myosin light chain kinase (20). Calmodulin, a highly conserved 17 kDa calcium sensor, adopts a dumbbell-like structure with two globular domains connected by a central α-helix, each domain containing two EF-hand calcium-binding motifs (21). Upon binding to the α-helical CBP, CaM undergoes a dramatic conformational change, wrapping around the peptide to form a compact globular complex (**Figure 1A**) (22). By genetically fusing CaM and CBP to the amino-terminal domains of antibody heavy and light chains, we engineered a modular peptide clamp capable of sterically occluding antibody paratopes. The resulting peptide clamp is linked to the antibody *via* a protease-cleavable sequence sensitive to matrix metalloproteinases (MMPs), particularly MMP-2 and MMP-9, which are frequently overexpressed in the tumor microenvironment. This design enables conditional, tumor-selective activation of the antibody, minimizing systemic toxicity and on-target, off-tumor effects (**Figure 1B**).

To validate our approach, we selected as a model for CaM-dependent functional blocking two clinically relevant monoclonal antibodies with known on-target, off-tumor toxicity: cetuximab (anti-EGFR) and trastuzumab (anti-HER2). Trastuzumab showed a 26% response rate in HER2-positive breast cancer patients (23). But in early trials, congestive heart failures were reported. Especially when patients were treated concurrently with trastuzumab and anthracyclines, cardiotoxic effects were observed in 27% of patients (24, 25). As a second example, we chose the monoclonal antibody cetuximab. In Phase II trials of cetuximab treatment in patients with epidermal growth factor receptor (EGFR) positive refractory colorectal cancer, more than 86% of patients developed acne-like skin rash, including 18% developing grade 3 symptoms (26). In healthy tissue, EFGR is commonly expressed in the basal layer of the epidermis and regulates growth, differentiation, and wound healing. By inhibiting EGFR functions, additional side effects arise as changes in the scalp and hair growth, xerosis and pruritus, as well as nail and eye disorders (27–29). In both breast cancer and colorectal cancer, overexpression of MMPs is reported, and increased MMP expression is associated with poor survival rates and tumor progression (30–32). CX-2051, an anti-EpCAM antibody-drug conjugate developed by CytomX Therapeutics using their PROBODY^®^ platform, is designed for protease-dependent activation within the tumor microenvironment. Recent interim results from Phase 1 dose escalation studies have demonstrated promising efficacy and a favorable safety profile (33).

In this study, we demonstrate the efficacy of our CaM-CBP peptide clamp in masking trastuzumab and cetuximab. We investigated the impact of linker length and peptide clamp orientation on masking efficiency, as assessed by on-cell binding and antibody-dependent cell-mediated cytotoxicity (ADCC) reporter assays. Masked antibodies exhibited significantly reduced binding and effector function, which was restored upon *in vitro* incubation with MMP-9. Additionally, we evaluated the thermal stability and aggregation propensity of masked constructs using fluorescence-based thermal shift assays and analytical size exclusion chromatography, respectively.

## 2 Materials and methods

### 2.1 Cell lines

SK-BR-3 and A431 cells were cultivated at 37°C and 5% CO_2_ and humid atmosphere in Dulbecco’s Eagle Medium (DMEM, Thermo Fisher Scientific) supplemented with 10% FBS (Merck Millipore, Burlington, MA, USA) and 1% Penicillin–Streptomycin (Sigma Aldrich, St. Louis, MI, USA) in T75 cell culture flasks. Sub-culturing was performed every 3–4 days. Expi293F™ cells (Gibco, Thermo Fisher Scientific: A14527) were cultivated at 37°C and 5% CO_2_ and humid atmosphere in Expi293™ Expression Medium (Thermo Fisher Scientific). Sub-culturing was performed every 3–4 days.

### 2.2 Cloning, protein expression and purification of masked antibodies

DNA coding for masked antibodies was cloned into pTT5 plasmids *via* Golden Gate assembly. Therefore, 100 ng of pTT5 plasmid was incubated with a 7-molar excess of insert, BsaI (NEB: R3733), and T4 DNA ligase (NEB: M0202). After the correct plasmid sequence was verified *via* sequencing, it was used for the transfection of Expi293F™ cells (Gibco, Thermo Fisher Scientific: A14527) following the manufacturer’s protocol, followed by purification *via* Protein A (1 mL HiTrap Protein A HP, Cytiva: 29048576) on an ÄKTA start chromatography system (Cytiva) as described before (34). Fractions were dialyzed against TBS buffer (150 mM NaCl, 50 mM TRIS pH 7.5).

### 2.3 Thermal shift assay

The thermal stability of antibodies was determined using a CFX Connect Real-Time PCR Detection System (Bio-Rad Laboratories GmbH). Briefly, 18 μL of protein solution (0.1-1 μg/μL) was mixed with 2 μL SYPRO^®^ Orange Protein Gel Stain (Sigma-Aldrich) diluted 1:10 with dH_2_O. Samples were measured in a Hard-Shell 96-well PCR plate. A temperature gradient of 0.5°C per minute was applied. Changes in fluorescence were measured in the FRET channel. The calculation of the thermal stability was performed using Bio-Rad CFX Manager Software version 3.0. Melting curves were visualized in GraphPad Prism 9.

### 2.4 On-cell binding assay

The on-cell EC_50_ of masked antibodies was determined by antibody titration using SK-BR-3 cells for masked trastuzumab and A431 cells for masked cetuximab. Therefore, cells were detached using Trypsin-EDTA (Gibco™) and washed once using PBS-B (0.1% BSA). Subsequently, 60.000 to 100.000 cells per well were transferred to a 96 well plate, washed once with PBS-B (0.1% BSA) and then incubated with antibodies in varying concentrations (333 nM to 0.05 nM, 1:3 serial dilution for trastuzumab constructs; 200 nM to 0.03 nM, 1:3 diluton for cetuximab constructs). The cells were incubated for 45 minutes on ice, afterwards washed twice using PBS-B, and then incubated with a 1:75 dilution of goat anti-human Fc PE secondary antibody (Thermo Scientific) for 15 minutes on ice. Afterwards, cells were washed three times before analysis by flow cytometry using a CytoFLEX S (Beckman Coulter). Binding curves were compiled from the geometric mean of the fluorescence in the PE channel and plotted in GraphPad Prism 9 using a sigmoidal 4-parameter logistic regression model to determine EC_50_ values. Fold reduction values were calculated by dividing the EC_50_ of masked antibodies by the EC_50_ of the unmodified parental antibody.

### 2.5 Antibody-dependent cell-mediated cytotoxicity assay

ADCC activity was assessed using the ADCC Reporter Bioassay Kit (G7010, Promega) according to the manufacturer’s guidelines. Briefly, 7,500 SK-BR-3 cells (for trastuzumab constructs) or A431 cells (for cetuximab constructs) were seeded per well in white, flat-bottom 96-well assay plates (Corning, Cat. #3917) and allowed to adhere by incubating for 20–24 h at 37°C with 5% CO_2_. Serial dilutions of antibody constructs were prepared (33 nM to 5 pM for trastuzumab; 10 nM to 1.5 pM for cetuximab; 1:3 serial dilution) and added to target cells. Effector cells were then introduced at effector-to-target cell ratios of 15:1 (trastuzumab) or 10:1 (cetuximab), followed by incubation for 6 h at 37°C and 5% CO_2_. Following incubation and addition of luciferin substrate solution, luminescence intensity was measured using a CLARIOstar Plus plate reader. Background luminescence, determined from a control well lacking antibody, was subtracted from each sample. The resulting luminescence values were plotted as a function of antibody concentration and analyzed by non-linear regression using a sigmoidal 4-parameter logistic model in GraphPad Prism 9 to determine the EC_50_. To quantify the impact of antibody masking, fold reduction values were calculated by dividing the EC_50_ of the masked antibody constructs by the EC_50_ of the corresponding unmodified parental antibody.

### 2.6 Size exclusion chromatography

The aggregation content of antibody samples was assessed using analytical SEC. Briefly, 13–20 μg of antibody was loaded onto a TSKgel SuperSW3000 column (Tosoh Bioscience) equilibrated in PBS (pH 7.4) using an Agilent 1260 Infinity HPLC system (Agilent Technologies). Chromatographic separation was performed at a constant flow rate of 0.35 mL/min, and protein elution was monitored by absorbance at 220 nm. SEC elution profiles were visualized in GraphPad Prism 9.

### 2.7 Demasking of antibodies by MMP-9 hydrolysis

To achieve demasking of antibodies, 0.1 μg of human MMP-9 (Acro Biosystems) was added per 0.1 mg of antibody, and the mixture was incubated at 37°C for 48 to 72 hours. Pro-MMP-9 was activated by incubation with 1 mM 4-aminophenylmercuric acetate (AMPA) overnight at 37°C. Following incubation of antibodies with MMP-9, the extent of proteolytic cleavage was assessed by SDS-PAGE under reducing conditions, allowing visualization of the released antibody fragments and confirming successful removal of the masking domain. The Color Prestained Protein Standard, Broad Range (10–250 kDa) (NEB) was used as a molecular weight marker.

### 2.8 Analysis of protein structures

For structural analysis, protein models were retrieved from the RCSB Protein Data Bank (PDB). All structures were visualized and analyzed using the built-in PDB viewer available through the RCSB PDB web interface. Distance measurements between selected residues were performed directly within the viewer’s measurement tool. Measurements were taken for the distance of the N-terminal domains of the heavy and light chain in the trastuzumab Fab (PDB: 6BGT), the distance of the N-terminal domains of the heavy and light chain in the cetuximab Fab (PDB: 1YY8), and the distance of the C-terminal domains of human CaM and CBP in the CaM-CBP complex (2LV6).

### 2.9 Isolation of PBMCs

Buffy coats from healthy donors were obtained from the German Red Cross Blood Donation Service Baden-Württemberg-Hessen, Frankfurt am Main, Germany. Density gradient centrifugation was used to isolate peripheral blood mononuclear cells, as previously described (35). All donors provided written informed consent, and the study was conducted in accordance with the Declaration of Helsinki. Distribution of immune cell populations was validated via flow cytometry. The following antibodies were utilized for staining: CD3-BUV395 (Clone: SK7), CD14-BV711 (Clone: M5E2), CD45-BV510 (Clone: HI30), and CD56-BV421 (Clone: NCAM16.2) (antibodies were obtained from BD Biosciences, San Jose, California, USA), CD19-BB515 (Clone: HIB19) and CD16-PE (Clone: 3G8) (both antibodies were obtained from BioLegend, San Diego, California, USA). Analysis of samples was carried out with the BD FACSCelesta instrument (36). For cryopreservation of PBMCs 5 x 10^6^ cells were collected by centrifugation (500 x g, 4 min) and resuspended in 500 µL medium A (RPMI 1640 GlutaMAX™ + 40% (v/v) FCS). Subsequently, 500 µL of medium B (RPMI 1640 GlutaMAX™ + 20% (v/v) DMSO) were added and the vial was gently inverted three times. The vials were transferred into a pre-cooled Mr. Frosty™ Freezing Container at 4°C, which was stored at -80°C for 24h. For long term storage, the cells were transferred into the vapor phase of liquid nitrogen.

### 2.10 PBMC-based killing assay

The PBMC-based killing assay was performed as described in Yamashita et al. (37). Briefly, A431 target cells were labeled with 5 μM carboxyfluorescein succinimidyl ester (CFSE, eBioscience) for 5 minutes, then seeded in growth medium into 96-well F-bottom culture plates and incubated for 24 hours at 37°C, 5% CO2. The following day, human PBMCs thawed from cryostock were added to the target cells at effector:target ratio of 7.5:1. Antibody constructs (cetuximab and variants) were added in a concentration range of 200 nM to 0.03 nM in RPMI 1640 supplemented with 10% heat-inactivated FBS and 1% PenStrep. Co-cultures were incubated for 24 hours at 37°C, 5% CO2. After incubation, cells were harvested and washed once, followed by staining with Fixable Viability Dye (FVD, eBioscience) for 20 minutes on ice in the dark. Cells were washed again and resuspended for flow cytometry analysis using a CytoFLEX S (Beckman Coulter). Target cells were gated based on CFSE positivity in the FITC channel, and dead target cells were identified via FVD staining in the APC-A750 channel.

## 3 Results

### 3.1 Rational design of a human-derived peptide clamp for antibody masking

To sterically block antigen binding, we engineered a peptide clamp fused to the amino terminus of the heavy and light chain of an antibody. By utilizing the combination of human CaM and CBP, we aimed to minimize immunogenicity issues (**Figure 1A**). The peptide clamp was connected to the antibody *via* MMP-2/-9 cleavable linkers (**Supplementary Table S1**). By introducing a peptide clamp with a MMP-2/-9 cleavable linker that imposes steric hindrance on antigen binding, the inhibition is subsequently reversed through proteolytic cleavage of the linker, thereby enabling selective restoration of antigen recognition predominantly within the tumor microenvironment (TME). This controlled demasking is anticipated to minimize off-target binding to healthy cells while enhancing specific interaction with tumor cells (**Figure 1B**). We hypothesized that the length of the linker connecting the antibody and the components of the peptide clamp could influence masking efficiency. Structural analysis of trastuzumab and cetuximab indicated that the distance between the amino termini of the V_H_ and V_L_ domains is approximately 34 Å (**Supplementary Figure S1**). In parallel, the distance between the carboxy termini of CaM and CBP in the CaM-CBP complex was calculated to be 41.12 Å (**Supplementary Figure S2A**), supporting the feasibility of positioning the clamp in close proximity to the paratope. To systematically investigate the impact of linker properties on masking efficiency, we designed two distinct linker variants: a 32 amino acid (aa) linker incorporating an MMP-2/-9 cleavage site flanked by two G_4_S repeat units (referred to as “long”), and a 12 aa linker with a single GS repeat flanking the cleavage site (referred to as “short” linker) (9, 38). We postulated that a shorter, less flexible linker would position the peptide clamp closer to the antibody, thereby increasing steric hindrance and enhancing masking efficacy. Furthermore, we considered that the orientation of the peptide clamp, specifically, the fusion of CaM or CBP to either the heavy or light chain, could influence masking efficiency due to the asymmetric molecular dimensions of the CaM-CBP complex. Projection of a line connecting the carboxy termini of CaM and CBP revealed that the complex is more voluminous near the carboxy terminus of CaM (**Supplementary Figure S2B**). Consequently, distinct masking effects were anticipated depending on the arrangement of the clamp components. To test these hypotheses, we generated a series of constructs for both trastuzumab and cetuximab. For each antibody, four variants were produced, differing in the orientation of the peptide clamp and the linker length: TrHC(CaM)LC(CBP)_long, TrHC(CaM)LC(CBP)_short, TrHC(CBP)LC(CaM)_long, and TrHC(CBP)LC(CaM)_short. The nomenclature reflects the antibody (Tr for trastuzumab), the fusion orientation with CaM fused to the heavy chain and CBP fused to the light chain (HC(CaM)LC(CBP)) or vice versa (HC(CBP)LC(CaM)), and the linker length (long or short). To assess the steric hinderance generated by only fusing CaM to the antibody, we also generated constructs in which only CaM was fused to either the heavy or light chain using the same linker variants: TrHC(CaM)_long, TrHC(CaM)_short, TrLC(CaM)_long, and TrLC(CaM)_short. To demonstrate the broad applicability of our approach, we transferred the same peptide clamp variants to cetuximab, resulting in the following constructs: CetHC(CaM)LC(CBP)_long, CetHC(CaM)LC(CBP)_short, CetHC(CBP)LC(CaM)_long, and CetHC(CBP)LC(CaM)_short. Additionally, we generated the corresponding CaM-only variants: CetHC(CaM)_long, CetHC(CaM)_short, CetLC(CaM)_long, and CetLC(CaM)_short. This comprehensive panel of constructs enabled a detailed evaluation of the contribution of linker properties, and clamp orientation to the efficiency of antibody masking.

### 3.2 Masking trastuzumab using the CaM-CBP-based peptide clamp

**Figure 2:**
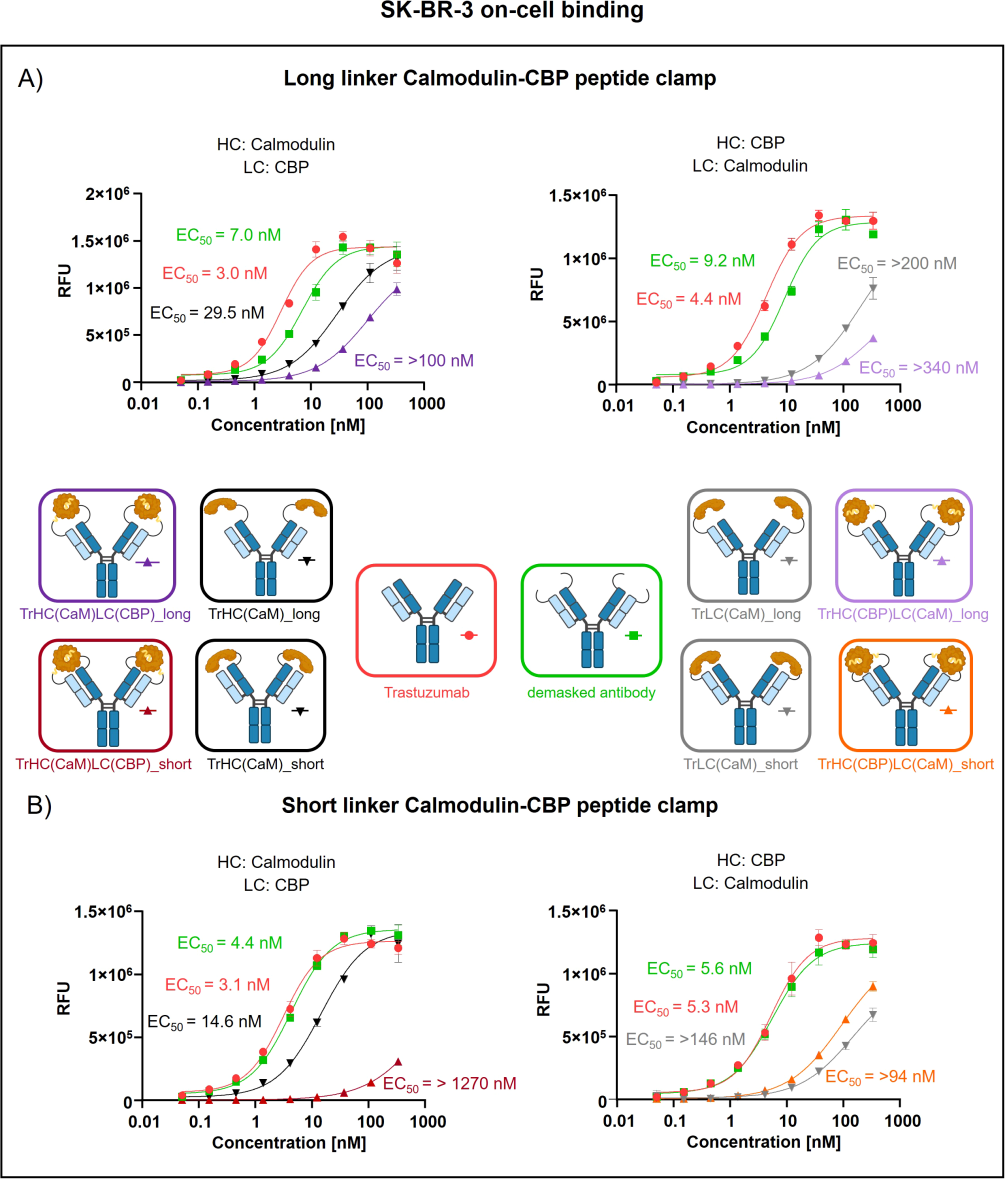
Evaluation of on-cell binding of trastuzumab masked with a calmodulin-CBP peptide clamp in different orientations. Analysis of concentration-dependent on-cell binding on HER2-positive SK-BR-3 cells using flow cytometry. Antibody concentration (nM) is plotted on the x-axis, and fluorescence intensity, measured in relative fluorescence units (RFU), is displayed on the y-axis. **(A)** Trastuzumab was masked using the CaM-CBP peptide clamp connected to the antibody *via* a long MMP-9 cleavable linker. The peptide clamp is engineered in two orientations: CaM fused to the N-terminus of the heavy chain and CBP to the N-terminus of the light chain (left), or the reverse configuration (right). **(B)** The linker used to connect trastuzumab and the peptide clamp was changed to a short MMP-9 cleavable linker. Moreover, the two orientations with CaM either fused to the N-terminus of the heavy chain and CBP to the N-terminus of the light chain (left), or the reverse configuration (right) was tested. Antibody binding was visualized using a PE-conjugated anti-human IgG detection antibody. Binding curves were fitted using a sigmoidal four-parameter logistic regression model. Error bars indicate the standard deviation of experimental triplicates. Experiments were repeated several times, showing similar results. Created with Biorender.com.

**Figure 3:**
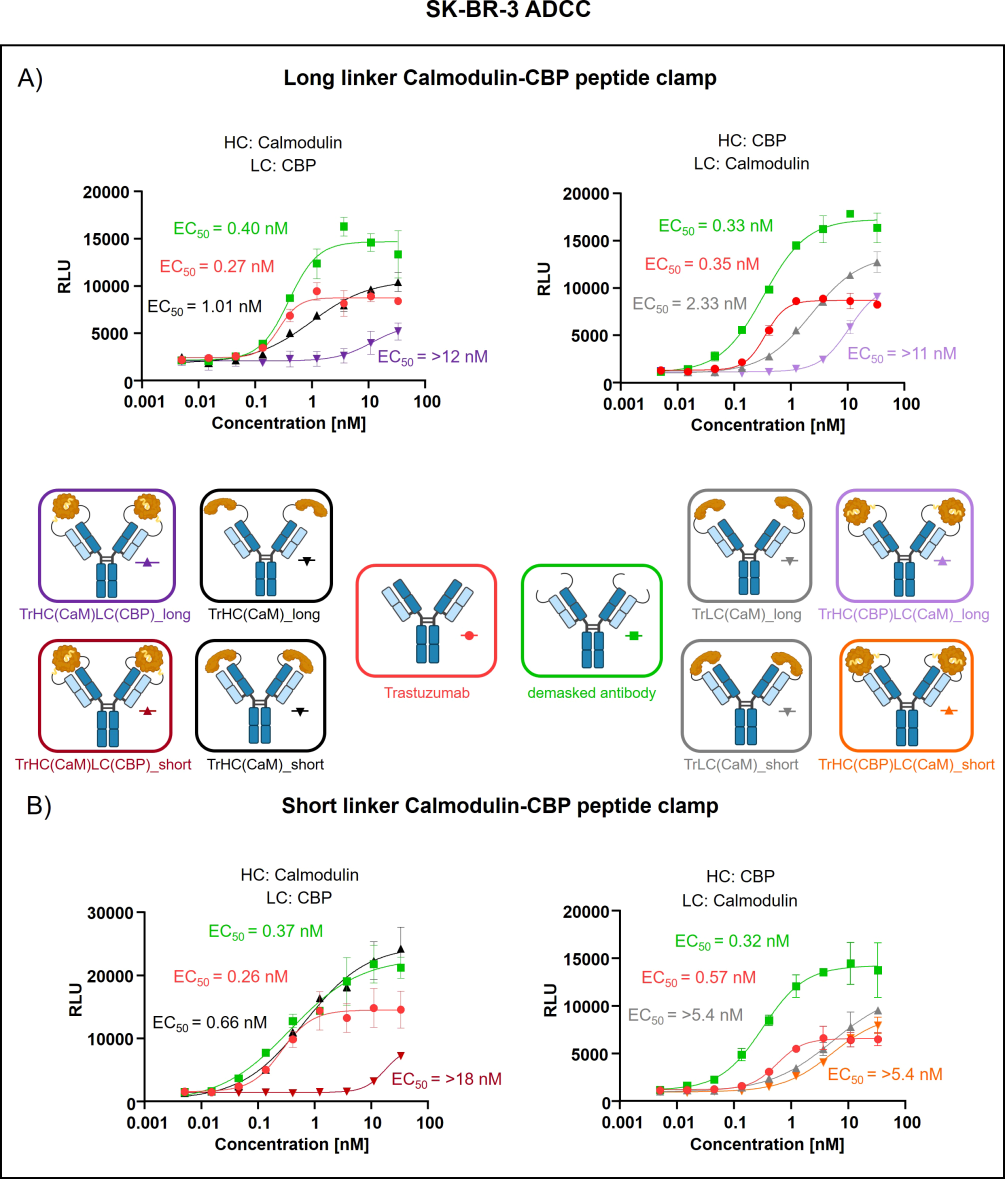
Evaluation of ADCC of trastuzumab masked with a calmodulin-CBP peptide clamp in different orientations. ADCC activity was assessed by a cell-based reporter assay utilizing HER2-overexpressing SK-BR-3 cells as target cells. Antibody concentration (nM) is plotted on the x-axis, and luminescence intensity, measured in relative luminescence units (RFU), is displayed on the y-axis. **(A)** By attaching the CaM-CBP peptide clamp to the N-terminal domains of trastuzumab via a long linker cleavable by MMP-9, the antibody was masked. The orientation of the peptide clamp was modified. Either CaM was fused to the N-terminus of the heavy chain and CBP to the N-terminus of the light chain (left), or vice versa (right). **(B)** By applying a short MMP-9 cleavable linker, the influence of the linker length on masking efficiency was tested. Again, the influence of the peptide clamp orientation on masking efficiency was tested by either fusing CaM to the N-terminus of the HC and CBP to the N-terminus of the LC (left), or fusion of the peptide clamp in the inverse orientation (right). Curves were fitted using a sigmoidal four-parameter logistic regression model. Error bars indicate the standard deviation of experimental duplicates. Experiments were repeated several times, showing similar results. Created with Biorender.com.

Peptide clamp-masked antibodies were produced in HEK293 cells, followed by Protein A purification. Analysis by analytical SEC was applied to examine aggregation behavior. Masked antibodies display low aggregation propensities with 3.9 to 7.6% aggregates, while unmodified trastuzumab displays an aggregation content of 1.3% (**Supplementary Figures S3A–D**). Next, we tested the influence of the peptide clamp on the thermal stability of the construct in a fluorescence-based thermal shift assay. For unmodified trastuzumab, three melting events can be observed at 64°C, 68°C, and 80°C. Peptide clamp-masked trastuzumab variants mostly display two melting events at Tm 61 to 65°C and 77 to 78°C (**Supplementary Figures S4A–D**). To simulate demasking in the TME, masked antibodies were incubated with MMP-9 *in vitro.* Peptide clamp removal was verified using SDS PAGE (**Supplementary Figures S5A–D**). All four antibody constructs exhibit shifts in the molecular weight of the heavy and light chains to molecular weights of the unmodified trastuzumab upon linker hydrolysis. Further, after cleavage, CaM was detected at a molecular weight of approximately 15 kDa or 21 kDa. After confirming linker cleavage, we investigated the blocking efficiency in an on-cell binding assay on SK-BR-3 cells (**Figure 2**). TrHC(CaM)LC(CPB)_long exhibited a 35-fold increase in EC_50_ compared to unmodified trastuzumab, with presence of CaM and CBP enhancing masking efficiency by approximately 3-fold comparing TrHC(CaM)LC(CBP)_long to TrHC(CaM)_long (**Figure 2A**). Upon MMP-9-mediated cleavage of the linker, antigen binding was restored to a level within 2-fold of the EC_50_ measured for unmodified trastuzumab. Altering the peptide clamp orientation to TrHC(CBP)LC(CaM)_long further increased masking efficiency, resulting in a 65-fold elevation in EC_50_ relative to trastuzumab. Again, presence of CaM and CBP increased masking efficiency, comparing the peptide clamp masked construct to the construct partially masked with only CaM fused to the light chain, and binding was restored after MMP-9-mediated linker cleavage. Decreasing linker length and flexibility (TrHC(CaM)LC(CBP)_short) further improved masking efficiency, with an over 257-fold increase in EC_50_ compared to trastuzumab and an 55-fold enhancement in masking efficiency relative to the corresponding non-clamp variant (**Figure 2B**). Whereas in the TrHC(CBP)LC(CaM)_short orientation, masking efficiency was decreased around 4-fold, and presence of CaM and CBP led to a decrease in masking efficiency. Upon linker cleavage, binding was restored to within 1.1- to 1.3-fold EC_50_ of that observed for unmodified trastuzumab. To investigate the additional steric hindrance introduced by an alpha-helical peptide not bound by calmodulin, we generated a construct in which CaM was fused to the heavy chain of trastuzumab via a short linker, and a 13-amino acid alpha-helical peptide was fused to the light chain of trastuzumab via a long linker, resulting in the construct TrHC(CaM)LC(helix)_short (**Supplementary Figure S6A**). Compared to TrHC(CaM)LC(CBP)_short, this construct exhibited more than a 2-fold reduction in masking efficiency (**Supplementary Figure S6B**, **Supplementary Table 2**). To assess whether reduced on-cell binding also translated to reduced effector function of peptide clamp-masked antibodies, we performed a reporter-cell-based ADCC assay using HER2-positive SK-BR-3 cells (**Figure 3**). Masked antibodies show significantly reduced ADCC. Trends in relative masking efficiency regarding linker length and peptide clamp orientation observed in on-cell binding were reflected in the ADCC assay. The construct TrHC(CaM)LC(CBP)_long showed a 25-fold increased EC_50_ (**Figure 3A**). While again TrHC(CaM)LC(CBP)_short displayed the greatest reduction in ADCC with a 43-fold increased EC_50_ (**Figure 3B**). Whereas trastuzumab induced maximal ADCC activation at 1 nM, TrHC(CaM)LC(CBP)_short required approximately 10 nM for detectable activation. Demasked antibodies, as well as antibodies only masked with CaM, and peptide clamp-masked antibodies exhibiting low masking efficiency, demonstrated enhanced maximum ADCC activation. Notably, the demasked TrHC(CBP)LC(CaM)_short variant exhibited a 2-fold higher E_max_ than unmodified trastuzumab at maximal activation. Importantly, this enhancement in maximal ADCC activation was not accompanied by a corresponding decrease in EC_50_ values.

### 3.3 Transferring the CaM-CBP-based peptide clamp to cetuximab

**Figure 4:**
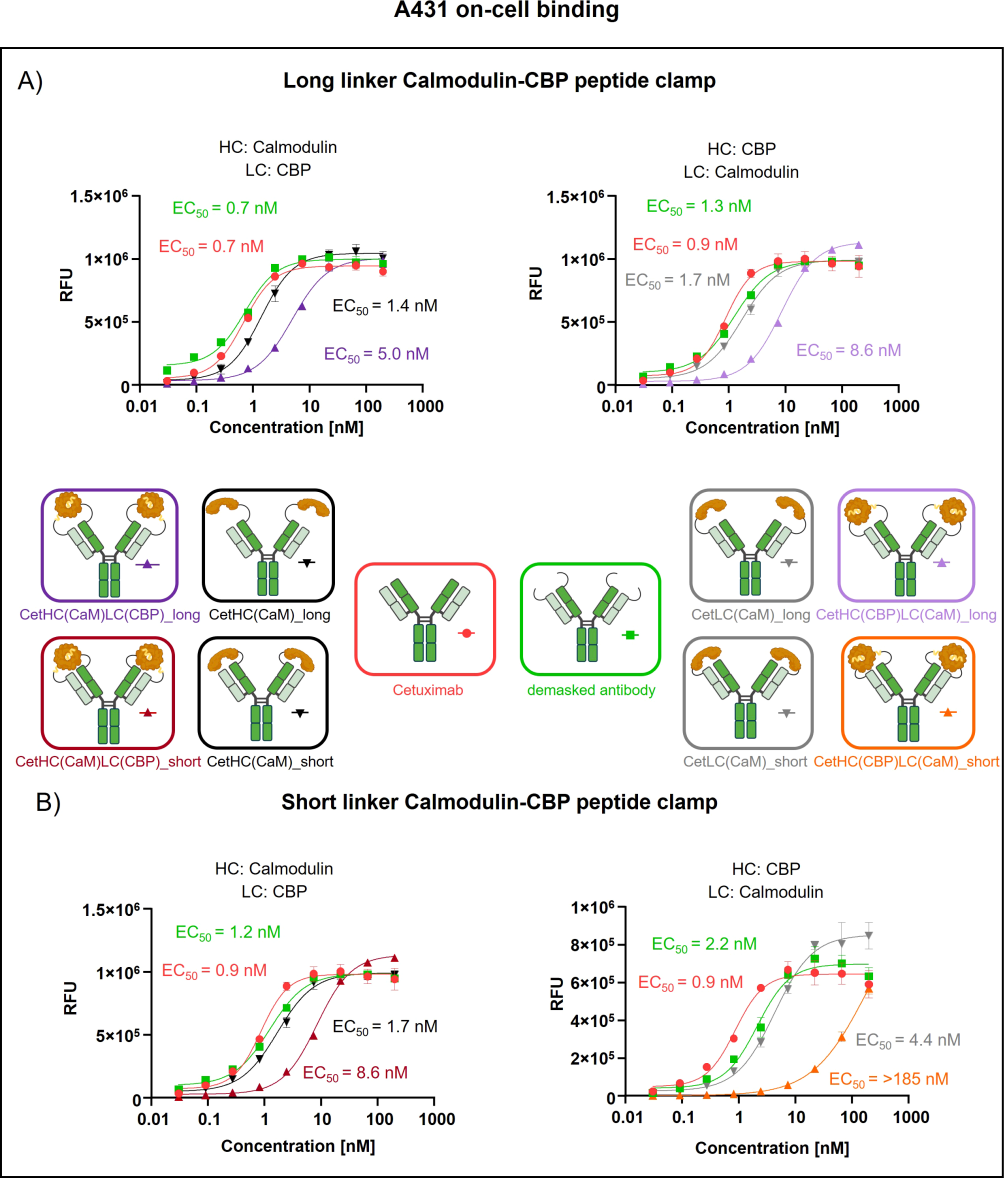
Evaluation of on-cell binding of cetuximab masked with a calmodulin-CBP peptide clamp in different orientations. Analysis of concentration-dependent on-cell binding on EGFR-positive A431 cells using flow cytometry. Antibody concentration (nM) is plotted on the x-axis, and fluorescence intensity, measured in relative fluorescence units (RFU), is displayed on the y-axis. **(A)** Cetuximab is masked using the CaM-CBP peptide clamp connected to the antibody *via* a long MMP-9 cleavable linker. The peptide clamp configuration is altered by fusing CaM to the N-terminus of the heavy chain and CBP to the N-terminus of the light chain (left) or employing the reciprocal arrangement (right). **(B)** The influence of the linker length connecting cetuximab and the peptide clamp is investigated by including a short MMP-9 cleavable linker. In addition, the influence of the peptide clamp orientation is tested by either fusing CaM to N-terminus of the heavy chain and CBP to N-terminus of the light chain (right) or the reverse configuration (left). Antibody binding is visualized using a PE-conjugated anti-human IgG detection antibody. Binding curves were fitted using a sigmoidal four-parameter logistic regression model. Error bars indicate the standard deviation of experimental triplicates. Experiments were repeated several times, showing similar results. Created with Biorender.com.

**Figure 5:**
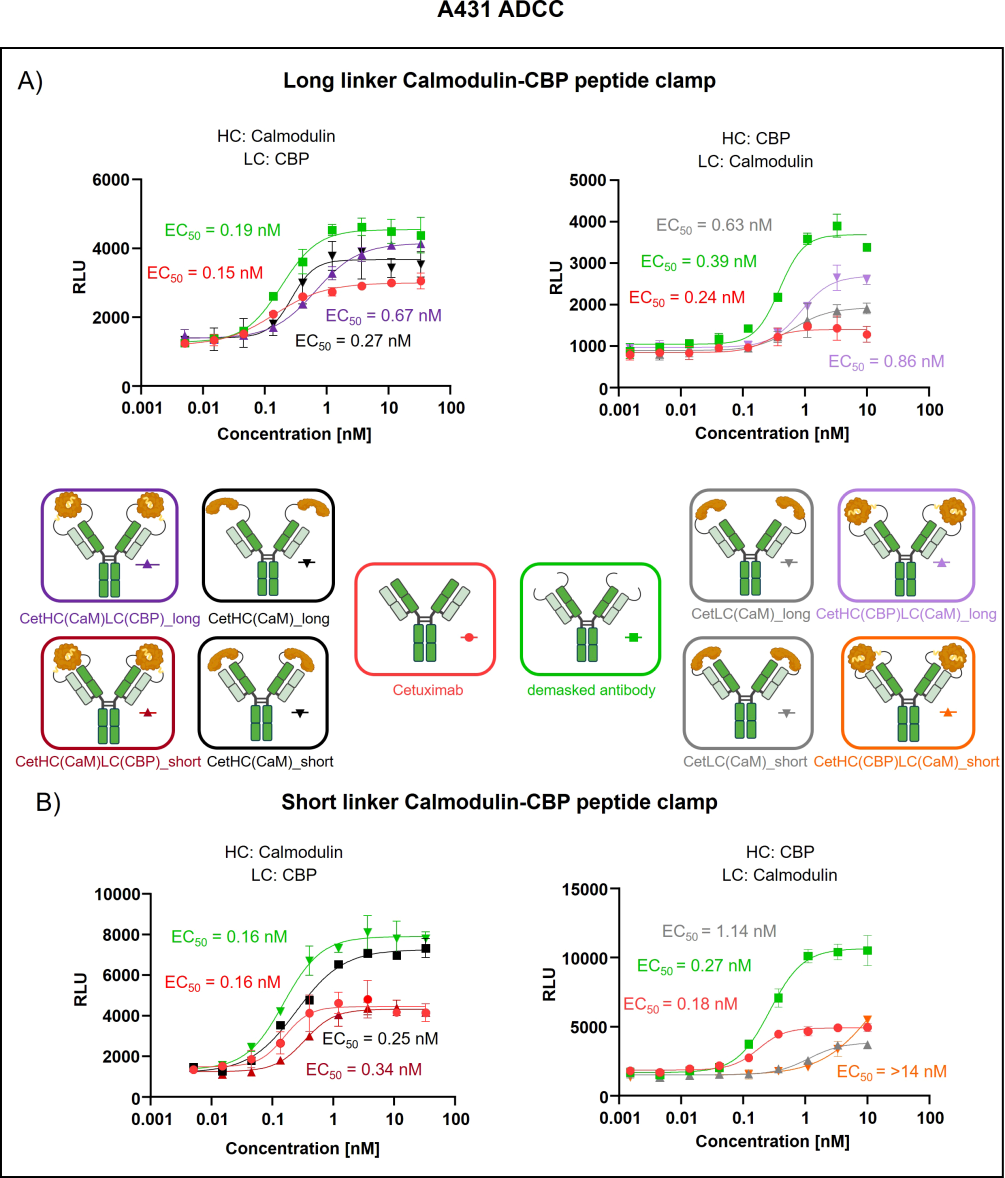
Evaluation of ADCC of cetuximab masked with a calmodulin-CBP peptide clamp in different orientations. ADCC was evaluated using a cell-based reporter assay with EGFR-positive A431 cells as the target cell line. Antibody concentration (nM) is plotted on the x-axis, and luminescence intensity, measured in relative luminescence units (RFU), is displayed on the y-axis. **(A)** Masking of cetuximab was achieved by connecting the CaM-CBP peptide clamp to the antibody *via* a long MMP-9 cleavable linker. The influence of the configuration of the peptide clamp on masking was assessed by either fusing CaM to the N-terminus of the heavy chain and CBP to the N-terminus of the light chain (left) or employing the reciprocal arrangement (right). **(B)** To assess the influence of the linker length connecting the antibody and the peptide clamp, a short MMP-9 cleavable linker is applied. Furthermore, CaM is either fused to N-terminus of the HC and CBP is fused N-terminus of the LC (left) or inversely (right). Curves were fitted using a sigmoidal four-parameter logistic regression model. Error bars indicate the standard deviation of experimental duplicates. Experiments were repeated several times, showing similar results. Created with Biorender.com.

To assess the generalizability of our peptide clamp masking strategy, we extended its application to cetuximab, a clinically relevant anti-EGFR monoclonal antibody. Analytical SEC revealed that masked cetuximab constructs exhibited low aggregation propensities, with aggregate content ranging from 3.9% to 6.8%, whereas unmodified cetuximab did not display detectable aggregation (**Supplementary Figures S3E–H**). Thermal stability analysis by fluorescence-based thermal shift assay revealed that unmodified cetuximab undergoes two distinct melting transitions at approximately 60°C and 72°C (**Supplementary Figure S4**). Masked cetuximab variants with CaM fused to the heavy chain exhibited melting transitions at 61-62°C and 67-68°C. In contrast, fusion of CaM to the light chain *via* a long linker resulted in transitions at 66°C and 72°C. Substitution of the long linker with a short linker in the light chain fusion yielded a single melting transition at 67°C. After confirming protein stability, proteolytic linker cleavage was achieved by *in vitro* incubation with MMP-9, as confirmed by SDS-PAGE analysis (**Supplementary Figures S5E–G**). Complete removal of the peptide clamp was observed, with demasked heavy and light chains migrating at positions consistent with unmodified cetuximab, while CaM was detected as a distinct band at approximately 21 kDa. On-cell binding assays using EGFR-positive A431 cells revealed that fusion of the peptide clamp *via* a long linker resulted in a modest 6-fold increase in EC_50_ independent on peptide clamp orientation (**Figure 4A**). Fusion of CaM alone to either the heavy or light chain resulted in a marginal 2-fold shift in EC_50_, regardless of orientation. Importantly, removal of the masking moiety restored binding of the antibody, with EC_50_ values only 1- to 1.4-fold higher than those of unmodified cetuximab. Substitution with the short linker and fusion of CaM to the light chain and CBP to the heavy chain (CetHC(CaM)LC(CBP)_short), yielded a 5-fold increase in EC_50_ relative to cetuximab, with binding largely restored following linker hydrolysis (1.2-fold EC_50_ compared to cetuximab; **Figure 4B**). Fusion of CaM solely to the heavy chain using the short linker resulted in a 1.5-fold increase in EC_50_. Strikingly, inverting the orientation of the peptide clamp with the short linker substantially enhanced masking efficiency. The construct CetHC(CBP)LC(CaM)_short demonstrated an EC_50_ increase of more than 140-fold compared to unmodified cetuximab. In contrast, fusion of CaM alone to the heavy chain with the short linker led to a 4-fold increase in EC_50_. Upon cleavage of the linker in CetHC(CBP)LC(CaM)_short, antigen binding was mostly restored, with EC_50_ remaining 7.2-fold higher than that of cetuximab. Functional validation of the observed effect was performed *via* an ADCC reporter assay utilizing EGFR-positive A431 target cells (**Figure 5**). For the majority of constructs evaluated, only a modest reduction in ADCC activation was detected, consistent with the trends observed in the on-cell binding assay. The construct CetHC(CaM)LC(CBP)_long only shows 5-fold higher EC_50_ compared to cetuximab (**Figure 5A**). Notably, the construct with the same orientation of the peptide clamp but connected with the short MMP-9 cleavable linker, CetHC(CBP)LC(CaM)_short, exhibited a pronounced impairment in ADCC activation, as evidenced by a greater than 78-fold increase in EC_50_ (**Figure 5B**). Cetuximab achieved maximal ADCC activation at approximately 0.4 nM, whereas CetHC(CBP)LC(CaM)_short required concentrations of around 1 nM to initiate detectable ADCC activation. Following linker hydrolysis, ADCC activation was restored, with only a 3-fold increase in EC_50_ relative to cetuximab. Fusion of CaM to the light chain *via* the short linker resulted in a 6-fold shift in EC_50_ and an accompanied decrease in maximal ADCC activation compared to cetuximab. Analyzing E_max_ values, an increase relative to unmodified cetuximab was observed for peptide clamp-masked antibodies with low masking efficiency (except CetHC(CaM)LC(CBP)_short), in partially masked antibodies containing only CaM fused to the antibody (except CetLC(CaM)_short), and in demasked antibodies. Notably, E_max_ was increased by up to 2.8-fold in demasked CetHC(CBP)LC(CaM)_long compared to unmodified cetuximab. To further assess the functional activity of CetHC(CBP)LC(CaM)_short, we evaluated its capacity to mediate ADCC in a PBMC-based killing assay of HER2-overexpressign A431 cells (**Supplementary Figure S7**). Consistent with the results obtained in the reporter cell-based ADCC assay, only minimal activity, was observed for the masked construct at concentrations around 10 nM, where maximal killing begins. Upon demasking, however, the antibody restored cytotoxic activity to levels comparable to unmodified cetuximab.

## 4 Discussion

In order to reduce on-target, but off-tumor side effects in cancer treatment using monoclonal antibodies, several masking strategies that rely on activation of the masked antibody in the tumor microenvironment have been proposed. For example, Adagene`s SAFEbody technology used to mask the anti-CTLA 4 antibody ADG126, showed promising results in Phase 1b/2 trials (39). Additionally, the steric-hinderance-based coiled-coil masking unit approach by Pfizer was used to generate the masked anti-CD47 antibody SGN-CD47M. Based on promising results in tolerability studies in cynomolgus monkeys and anti-tumor activity in xenograft mouse models, this therapeutic has recently advanced into phase I dose-escalation clinical trials (19).

While generic, steric hindrance-based masking strategies have demonstrated robust masking efficiencies, the use of non-human masking moieties raises concerns regarding potential immunogenicity (11, 18). In this study, we introduce a novel, potentially generic, and fully human peptide clamp masking strategy based on the CaM-CBP interaction. This system enables conditional antibody activation *via* tumor-associated protease cleavage (MMP-9), thereby restoring antigen binding selectively in a conditional manner. We demonstrate that the peptide clamp can be modularly transferred between two different therapeutic antibodies. Utilizing rational design, we investigated the influence of linker length on masking efficiency. When applied to trastuzumab, the masking strategy resulted in a 257-fold reduction in on-cell binding EC_50_ on HER2-positive SK-BR-3 cells (**Supplementary Table S2**). Similarly, transferring the mask to cetuximab yielded a 140-fold decrease in on-cell binding EC_50_ on EGFR-positive A431 cells. Our data further indicate that masking efficiency is influenced by both the orientation and the linker length of the peptide clamp. Functionally, the reduction in on-cell binding correlated with diminished effector function. In a reporter cell-based antibody-dependent cellular cytotoxicity (ADCC) assay, masked trastuzumab and cetuximab exhibited 43-fold and 78-fold reductions in ADCC activation, respectively. Notably, at concentrations corresponding to the onset of maximal ADCC activation by unmodified antibodies, the masked variants did not elicit detectable ADCC activity. *In vitro* removal of the masking moiety *via* MMP-9-mediated hydrolysis resulted in the restoration of both on-cell binding and ADCC effector function to levels comparable to those observed with the unmodified antibody.

When investigating the influence of the linker length connecting the peptide clamp and the orientation of the peptide clamp, we found fusion of CaM and CBP to the antibodies only mildly increased aggregate formation by up to 6.8%. The content of aggregates was around 2-fold higher when CaM was fused to the heavy chain of the antibody (**Supplementary Figure S3**). Aggregate formation could be due to the homodimerization of masked antibodies when CaM on one antibody binds the CBP on another antibody in proximity. In general, masked antibodies displayed decreased retention time compared to the unmodified antibody. This is most likely caused by the increase in hydrodynamic radius due to the peptide clamp. All SEC chromatograms exhibited a peak at around 12 minutes, including the unmodified antibodies, suggesting an artifact possibly attributable to buffer components such as TRIS, despite their expected elution at a later timepoint. Following MMP-9 cleavage of the linker, the amount of aggregates decreases, and the retention time is similar to the unmodified antibody (**Supplementary Figure S8**). Next, melting temperatures were determined. For unmodified trastuzumab, three melting events were observed at 64°C, 68°C, and 80°C (**Supplementary Figure S4**). In a structural investigation of IgG1 antibodies, it was reported that melting of the Fc fragment results in two transitions with melting temperatures around 66°C and 82°C corresponding to melting of the CH2 and CH3 domain, while melting of the Fab fragment results in one transition with a Tm around 70°C (31). Fusing the peptide clamp to trastuzumab and cetuximab slightly decreased the thermal stability of the antibodies by a maximum of 7°C. The strongest decrease in stability was seen in the second melting event for masked trastuzumab variants, probably corresponding to the stability of the Fab region. While unmodified showed a Tm of 68°C, masked variants showed 67°C to 61°C, with some displaying overlapping melting of the CH2 domain and Fab. The decrease in thermal stability could be induced by disrupted domain packing or weakening stabilizing interactions at the heavy and light chain interface introduced by fusion of the peptide clamp to the antibody. Upon MMP-9 cleavage, Tm values did not change, indicating a structural difference in the Fab region. The decrease in thermal stability should not hinder developability, as in developability case studies, an IgG antibody is considered to display good thermodynamic stability with an onset of thermal transition at 56°C (40). Following demasking, SDS-PAGE analysis of the antibodies revealed expected shifts in the apparent molecular masses of both the heavy and light chains, consistent with those observed for the unmodified antibody (**Supplementary Figure S5**). CaM was detected at a molecular weight of approximately 15 kDa or 21 kDa, resulting from different folding states that could arise from deviations in incubation time at 98°C before SDS PAGE, as shown previously (31).

Not only does the orientation and linker length of the peptide clamp slightly influence aggregation behavior and thermal stability, but it also affects masking efficiency. Modifying TrHC(CaM)LC(CBP)_long, decreasing the linker length, strongly enhanced masking efficiency (**Figures 2A, B**, **3A, B**). In contrast, decreasing the linker length with the orientation of the peptide clamp switched (TrHC(CBP)LC(CaM)_long), decreased masking efficiency (**Figures 2A, B**, **3A, B**). Investigating the masking of cetuximab, only CetHC(CBP)LC(CaM)_short showed efficient masking (**Figures 4B**, **5B**). In both trastuzumab and cetuximab variants, constructs featuring the shortest linker exhibited the greatest extent of antigen masking. Notably, for trastuzumab, the variant with CaM fused to the heavy chain and CBP fused to the light chain exhibited the greatest extent of masking, while for cetuximab, the variant with CaM fused to the light chain and CBP fused to the heavy chain exhibited the greatest extent of masking. Differences in masking efficiency could result from the positioning of the peptide clamp relative to the complementarity-determining regions (CDRs) of the antibody. As the CaM-CBP complex is asymmetric in molecular mass near the carboxy termini of CaM and CBP, changing the orientation of the peptide clamp influences the steric hindrance induced in the antibody-antigen interaction (**Supplementary Figure S2B**). The distance and positioning of the N-termini to the main interaction points in the antibody-antigen interaction could further influence the masking efficiency. In both the trastuzumab-HER2-complex and the cetuximab-EGFR-complex, the distance from the N-termini of the light chains to the CDR3 of the heavy and light chains is shorter than the distance from the N-termini of the HC to the CDR3s (**Supplementary Figure S9**). For both antibodies, the CDR3 mainly contributes to antigen-binding (41, 42). In performing reporter-cell-based ADCC assays, we showed, as expected, that reduced on-cell binding also leads to reduced ADCC activation. The reduction in EC_50_ upon masking observed in on-cell binding studies was stronger than in the ADCC assay. This could result from differences in assay sensitivity. While in on-cell binding, every bound antibody leads to a signal, the ADCC assay relies on pathway activation. Already, a small number of bound antibodies can trigger effector cell pathway activation, thereby increasing sensitivity. Analyzing demasked antibodies, we saw an increase in E_max_ compared to unmodified antibodies. This phenomenon could have different reasons. It could arise from increased k_off_ of demasked antibodies resulting from linker residues still left after cleavage. By faster k_off,_ antibodies could increase the local abundance of the effector cells near the cancer cell due to a shorter time of interaction. Another explanation could be increased calcium concentration by CaM. The reporter cell-based ADCC assay quantifies activation of the nuclear factor of activated T-cells (NFAT) pathway by employing a gene reporter system in which the NFAT response element drives luciferase expression. Activation of the NFAT pathway relies on an intracellular increase in Ca^2+^ concentration, leading to activation of Calcineurin, a phosphatase that dephosphorylates NFAT proteins (43). This increase in intracellular Ca^2+^ is achieved by depletion of calcium stores in the endoplasmic reticulum (ER), followed by calcium influx from the extracellular space through store-operated calcium channels. Internalization of antibodies containing CaM *via* receptor-mediated endocytosis could increase the Ca^2+^ concentration in the ER. Following lysosomal degradation of these complexes, liberated Ca^2+^ might further amplify the intracellular Ca^2+^ signal, followed by potentially enhanced NFAT pathway activation. When testing the CetHC(CBP)LC(CaM)_short in a PBMC-based killing assay, the enhanced E_max_ was not observed, pointing toward a bias in the reporter cell based assay (**Supplementary Figure S8**).

We showed that the peptide clamp is transferable between antibodies by efficiently masking trastuzumab (**Figures 2**, **3**) and cetuximab (**Figures 4**, **5**). Therefore, this newly developed masking strategy could potentially be transferable to mask any antibody. To test this, the peptide clamp should be applied to a broader range of therapeutic antibodies. As we showed, finding the optimal orientation and linker length of the mask is critical; therefore, building a tool to predict the ideal orientation and linker length on each antibody could be cost-saving and time-saving. However, such a tool remains to be established. When incorporating the CaM-CBP peptide clamp into antibody constructs, we recommend using the short linker to connect the peptide clamp to the antibody. Additionally, it is advisable to empirically test both orientations of the clamp attachment to identify the variant that provides the most effective masking of antibody-antigen interaction. Previously, using molecular dynamics simulations, Chen et al. showed that the relative positioning of a steric-hinderance-based mask to the antibody CDRs strongly influences masking efficiency (44). Demasking of antibodies restored target binding. But the kinetics of the demasking reaction and the influence of the linker length and MMP-9 cleavage sequence are still to be determined. We propose that native mass spectrometry represents a robust analytical platform for investigating these processes. Specifically, by in-line activation of inactive pro-MMP-9 through the addition of 4-aminophenylmercuric acetate (APMA), followed by real-time monitoring of immunocomplex formation between the unmasked antibody and its antigen at defined time intervals, it should be possible to interrogate the kinetics of linker hydrolysis (45, 46). By now, only *in vitro* experiments have been performed, but to further characterize linker stability and, most importantly, demasking of peptide clamp-masked antibodies in the TME, *in vivo* studies should be considered. Recently, in animal studies, it was shown that steric-hinderance-based masking enhances the tolerability of antibodies with on-target, but off-tumor side effects. Furthermore, after 72 hours post-dose, 50% of the masked antibody is found demasked in the tumor (19). The demasking of the antibody in the TME remains one major limitation of masked antibodies. Whether our approach can achieve efficient demasking within the TME remains to be determined and should be addressed in future studies, for example using tumor graft models. Additionally, as we concluded for TrHC(CaM)LC(CBP)_short and CetHC(CBP)LC(CaM)_short, formation of the peptide clamp can be necessary for efficient masking, and only fusing CaM to the heavy chain of trastuzumab using the short linker did not result in efficient masking. Furthermore additionally fusing an alpha-helical peptide to the N-terminus of the light chain using the long MMP-9 cleavable linker TrHC(CaM)LC(helix)_short) lead to 2-fold lower EC_50_ compared to TrHC(CaM)LC(CBP)_short (**Supplementary Figure S7**). Whether the observed change in masking efficiency is due to peptide-clamp formation or additional steric hindrance remains unclear. At present, we lack direct evidence for peptide-clamp formation. Future studies should therefore aim to directly demonstrate such interactions, for example by employing native mass spectrometry or high-resolution structural approaches such as cryo-EM. Converting the peptide clamp into a logic gate approach by utilizing different stimuli for linker cleavage could be conceivable. This could be especially useful for bispecific antibodies, where each binding site is restored under certain conditions. Another topic to be investigated is whether the CaM-CBP peptide clamp can be applied to antibody-drug conjugates. In conclusion, we have developed a novel and promising masking strategy based on a human CaM-CBP peptide clamp. This approach offers the advantage of potentially being generic and, due to its human-derived components, is unlikely to elicit immunogenicity. However, further studies, particularly *in vivo* studies, are required to validate this strategy and to explore its full range of potential applications. Additionally, the CaM–CBP peptide clamp has so far only been evaluated on two therapeutic antibodies, where it showed efficient blocking. To establish whether this strategy is generically applicable, further studies should explore its masking capacity across a wider range of antibodies.

## Data Availability

The original contributions presented in the study are included in the article/**Supplementary Material**. Further inquiries can be directed to the corresponding author.
