# Supplementary material for: A masking clamp for conditional activation of therapeutic antibodies
Source: Front Immunol. 2025 Oct 30;16:1640427. doi: 10.3389/fimmu.2025.1640427 (PMC12611947; doi:10.3389/fimmu.2025.1640427)
Supplement: Supplementary file 1 [file DataSheet1.docx]

Supplementary Material

**A Masking clamp for conditional activation of therapeutic antibodies**

Adrian Bloch, Jan Zimmermann, Jan Habermann, Evelyn Ullrich, Harald Kolmar

# Supplementary Tables

**Supplementary Table 1. Protein sequence of MMP-9 cleavable linkers.** The MMP-9 cleavage site is indicated in red letters.

| Linker type | Sequence | Length [aa] |
| --- | --- | --- |
| Long | GGGGSGGGGSVHMPLGFLGPGGGGSGGGSGG | 32 |
| Short | GSVPLSLYSGGS | 12 |

**Supplementary Table 2. Characterization of peptide clamp-masked antibodies.** Summarized findings regarding binding behavior and physical properties of masked antibodies. EC_50_ values were calculated using GraphPad Prism 9 using a sigmoidal four-parameter logistic regression model. Fold EC_50_ values were calculated by dividing the EC_50_ of masked antibodies by the EC_50_ of the corresponding unmodified antibody. Values are shown as the mean of two or three measurements with standard deviation. *EC50 can only be determined in two of three experiments in the measured concentration due to low target binding. **experiments were repeated twice (n=2)***experiments were repeated three times (n=3).

| Construct | Cell-Binding fold EC_50_ | ADCC fold EC_50_ | Melting temp. [°C] | Aggregates [%] |
| --- | --- | --- | --- | --- |
| Trastuzumab | 1.0 | 1.0 | 64; 68; 80 | 1.3 |
| TrHC(CaM)LC(CBP)_long | 34.8 ± 1.8*** | 24.5 ± 13.1*** | 65; 78 | 7.6 |
| TrHC(CaM)LC(CBP)_long MMP-9 cleaved | 2.1 ± 0.2*** | 1.4 ± 0.1*** | 67; 78 | 2.7 |
| TrHC(CaM)_long | 9.8 | 3.7 | – | – |
| TrHC(CBP)LC(CaM)_long | 65.1 ± 13.8** | 22.4 ± 7.3** | 63; 67; 77 | 4.7 |
| TrHC(CBP)LC(CaM)_long MMP-9 cleaved | 2.0 ± 0.2** | 0.8 ± 0.2** | 63; 67; 77 | – |
| TrLC(CaM)_long | 36.1 ± 12.0** | 5.6 ± 0.9** | – | – |
| TrHC(CaM)LC(CBP)_short | >257 ± 153* | > 43 ± 18*** | 63; 78 | 7.4 |
| TrHC(CaM)LC(CBP)_short MMP-9 cleaved | 1.3 ± 0.1*** | 1.0 ± 0.3*** | 63; 78 | – |
| TrHC(CaM)_short | 4.7 ± 0.0** | 2.5 | – | – |
| TrHC(CBP)LC(CaM)_short | 13.3 ± 4.4** | 7.1 ± 2.4** | 61; 79 | 3.9 |
| TrHC(CBP)LC(CaM)_short MMP-9 cleaved | 1.1 ± 0.0** | 0.6 ± 0.0** | 61; 78 | – |
| TrLC(CaM)_short | 27.8 ± 0.3** | 6.5 ± 3.1** | – | – |
| TrHC(CaM)LC(helix)_short | 123 ± 69** | – | – | – |
| TrHC(CaM)LC(helix)_short MMP-9 cleaved | 1.7 ± 0.1** | – | – | – |
| Cetuximab | 1.0 | 1.0 | 60; 72 | 0 |
| CetHC(CaM)LC(CBP)_long | 6.0 ± 1.2** | 5.5 ± 1.1** | 61; 68 | 5.7 |
| CetHC(CaM)LC(CBP)_long MMP-9 cleaved | 1.0 ± 0.0** | 1.4 ± 0.1** | 61; 71 | – |
| CetHC(CaM)_long | 2.2 ± 0.2** | 2.2 ± 0.4** | – | – |
| CetHC(CBP)LC(CaM)_long | 6.6 ± 3.0** | 3.5 ± 0.1** | 66; 72 | 3.9 |
| CetHC(CBP)LC(CaM)_long MMP-9 cleaved | 1.4 ± 0.0** | 1.5 ± 0.2** | 66; 72 | – |
| CetLC(CaM)_long | 1.9 ± 0.1** | 2.3 ± 0.4** | – | – |
| CetHC(CaM)LC(CBP)_short | 5.4 ± 1.8** | 2.3 ± 0.1** | 62; 67 | 6.8 |
| CetHC(CaM)LC(CBP)_short MMP-9 cleaved | 1.2 ± 0.2** | 0.9 ± 0.1** | 61; 67 | – |
| CetHC(CaM)_short | 1.5 ± 0.2** | 1.6 ± 0.0** | – | – |
| CetHC(CBP)LC(CaM)_short | >140 ± 46*** | >78 ± 45*** | 67 | 3.9 |
| CetHC(CBP)LC(CaM)_short MMP-9 cleaved | 7.2 ± 3.6*** | 3.0 ± 2.2*** | 67 | – |
| CetLC(CaM)_short | 3.8 ± 0.8*** | 6.1 ± 0.3*** | – | – |

# Supplementary Figures


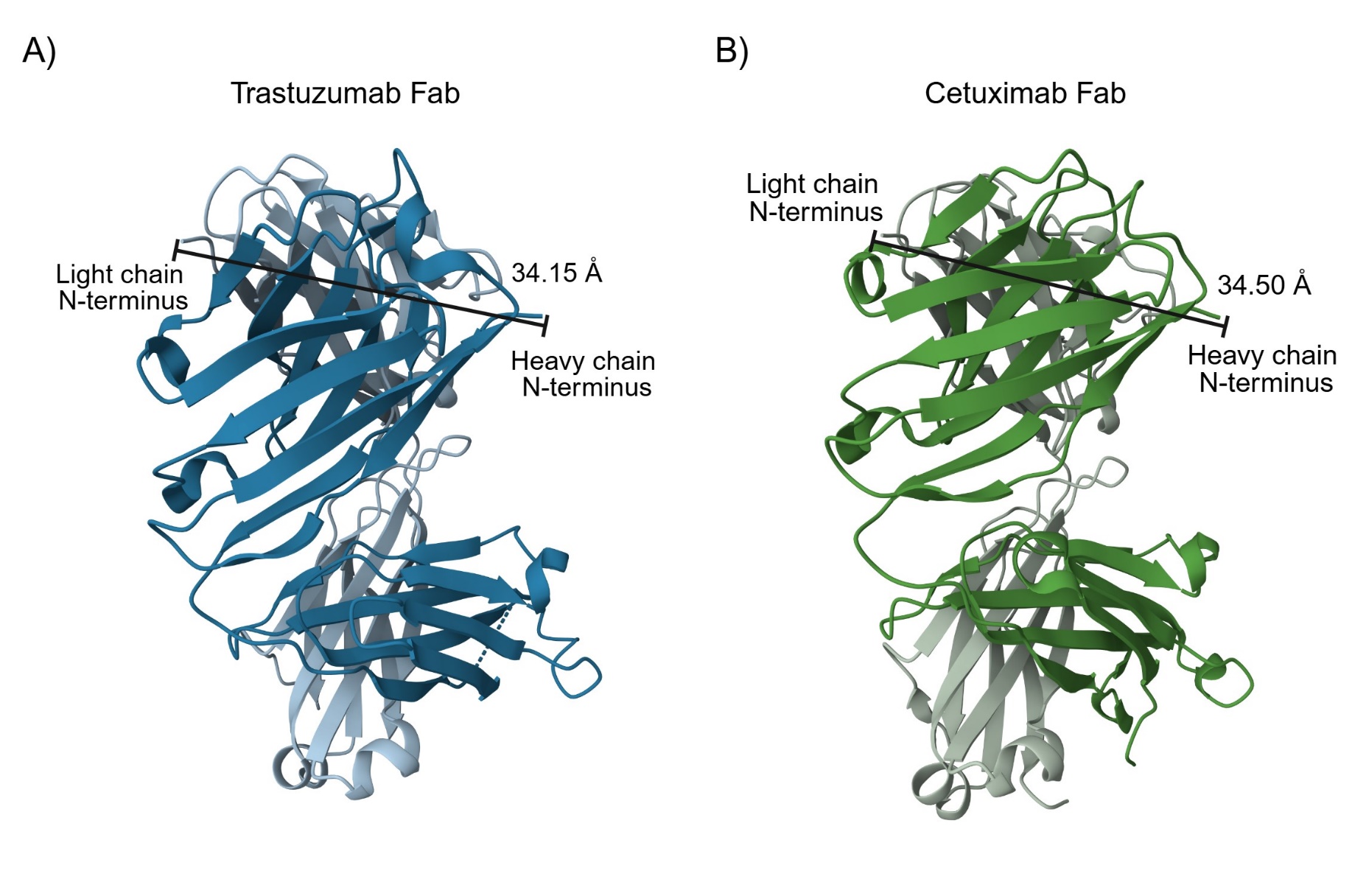


**Supplementary Figure 1: Distance measurement of the N-termini of the trastuzumab and cetuximab heavy and light chains. (A)** The trastuzumab heavy chain is shown in dark blue, and the light chain is shown in light blue. A black line indicates the distance between the N-terminal domains. Structure adapted from PDB: 6BGT. **(B)** The cetuximab heavy chain is shown in dark green, and the light chain is shown in light green. A black line indicates the distance between the N-terminal domains. Structure adapted from PDB: 1YY8.


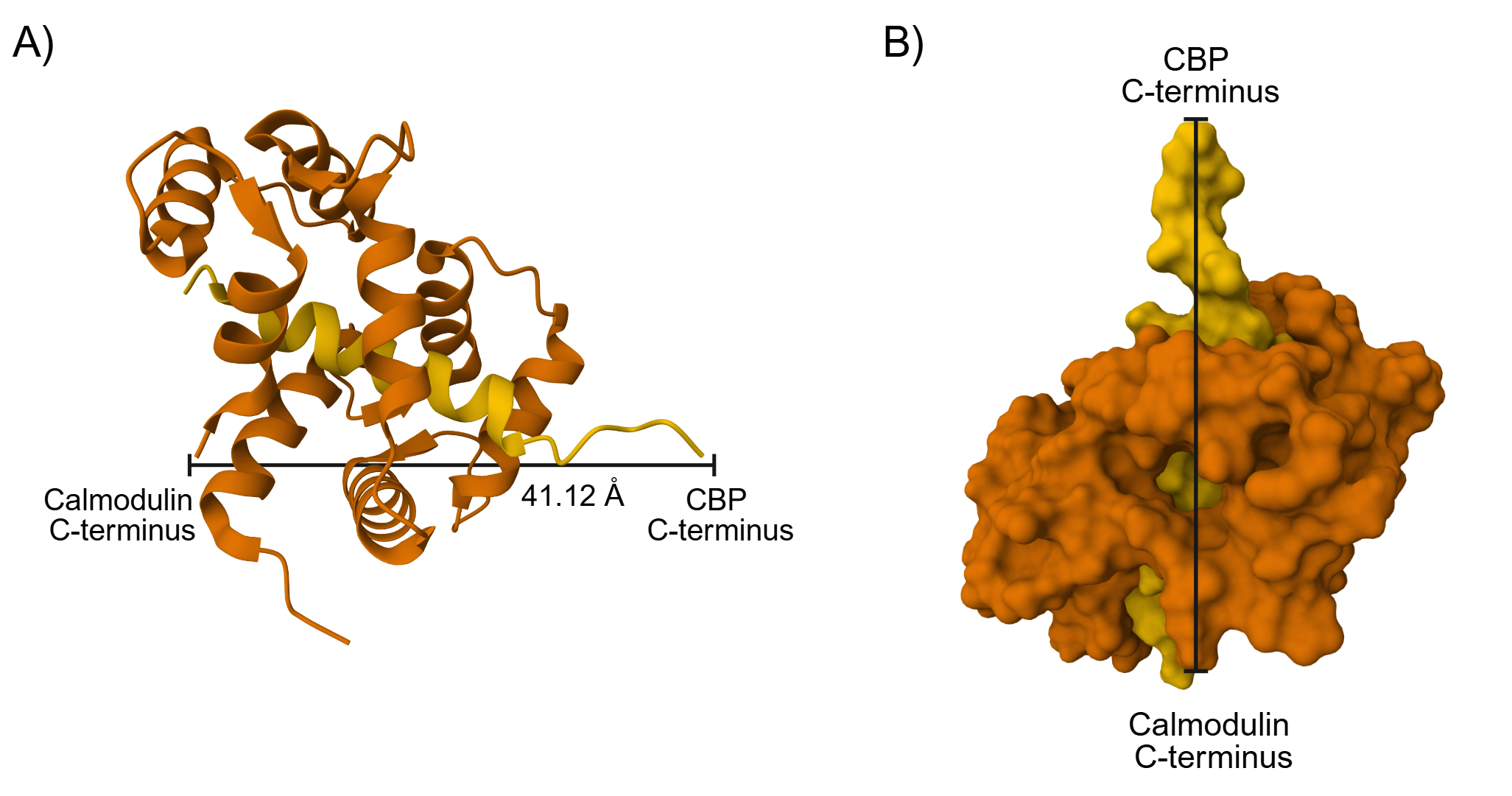


**Supplementary Figure 2. Distance measurement of the C-terminal domains of Calmodulin and CBP. (A)**Calmodulin is depicted in orange, and CBP is shown in yellow. A black line indicates the distance between the C-terminal domains. **(B)** Visualization of the distribution of the volume of the calmodulin-CBP complex along the axis connecting the two C-termini of the proteins. Structure adapted from PDB: 2LV6.


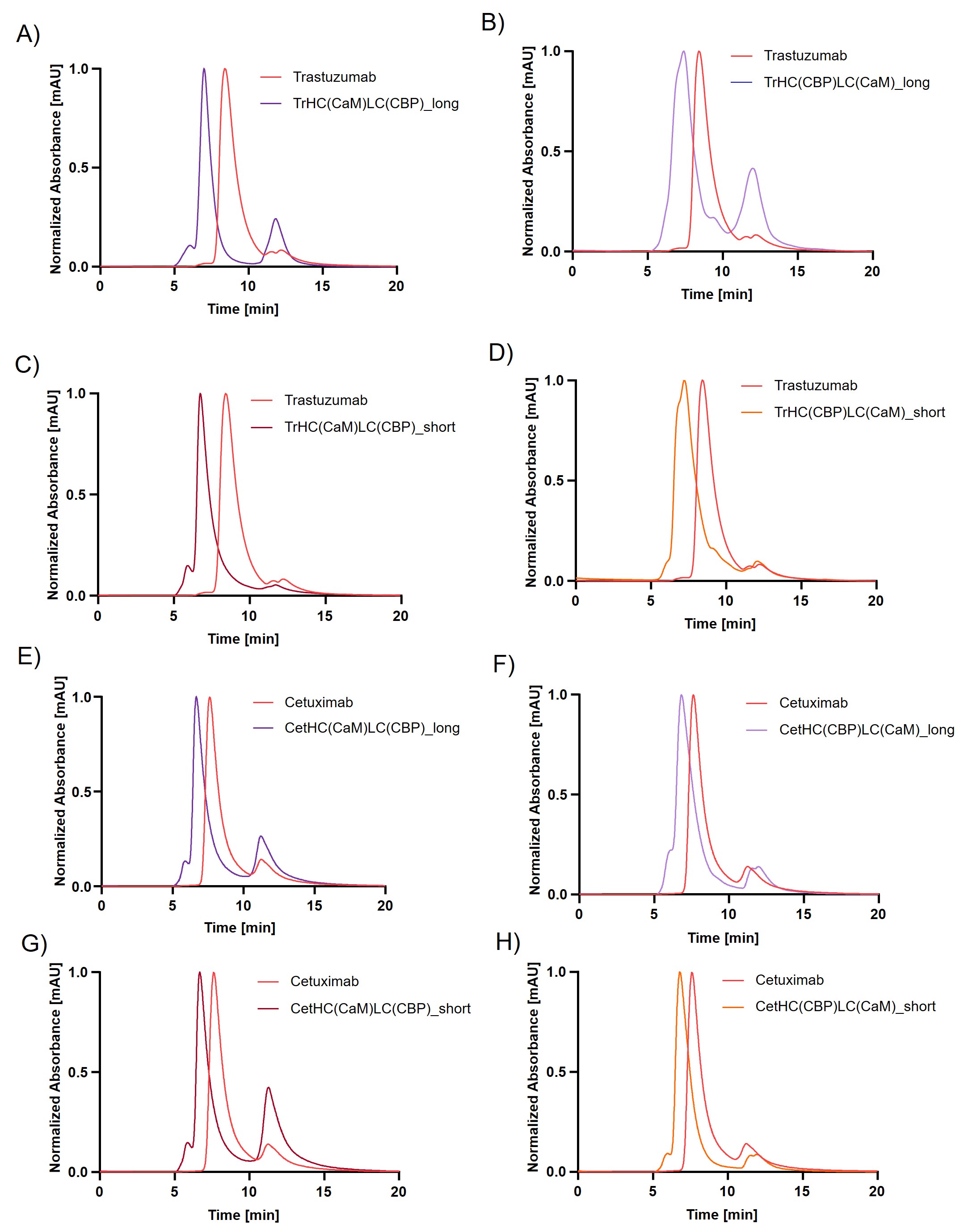


**Supplementary Figure 3. Analysis of aggregation propensity of peptide clamped-masked antibodies by analytical size exclusion chromatography.** The time in minutes is shown on the x‑axis, and normalized absorbance in mAu is shown on the y-axis. The unmodified trastuzumab or cetuximab is displayed in red, while the antibody masked with the peptide clamp is displayed in individual colors for each masked construct. **(A)** Analytical SEC of the trastuzumab construct with CaM fused to the V_H_, and CBP fused to V_L_ *via* the long MMP-9‑cleavable linker. **(B)** Analytical SEC of the trastuzumab construct with CBP fused to the V_H_,and CaM fused to V_L_ *via* the long MMP-9‑cleavable linker. **(C)** Analytical SEC of the trastuzumab construct with CaM fused to the V_H_, and CBP fused to V_L_ *via* the short MMP-9‑cleavable linker. **(D)** Analytical SEC of the trastuzumab construct with CBP fused to the V_H_,and CaM fused to V_L_ *via* the short MMP-9-cleavable linker. **(E)** Analytical SEC of the cetuximab construct with CaM fused to the V_H_,and CBP fused to V_L_ *via* the long MMP-9‑cleavable linker. **(F)** Analytical SEC of cetuximab construct with CBP fused to the V_H_, and CaM fused to V_L_ *via* the long MMP-9‑cleavable linker. **(G)** Analytical SEC of the cetuximab construct with CaM fused to the V_H_,and CBP fused to V_L_ *via* the short MMP-9‑cleavable linker. **(H)** Analytical SEC of the cetuximab construct with CBP fused to the V_H_,and CaM fused to V_L_ *via* the short MMP-9‑cleavable linker.

**
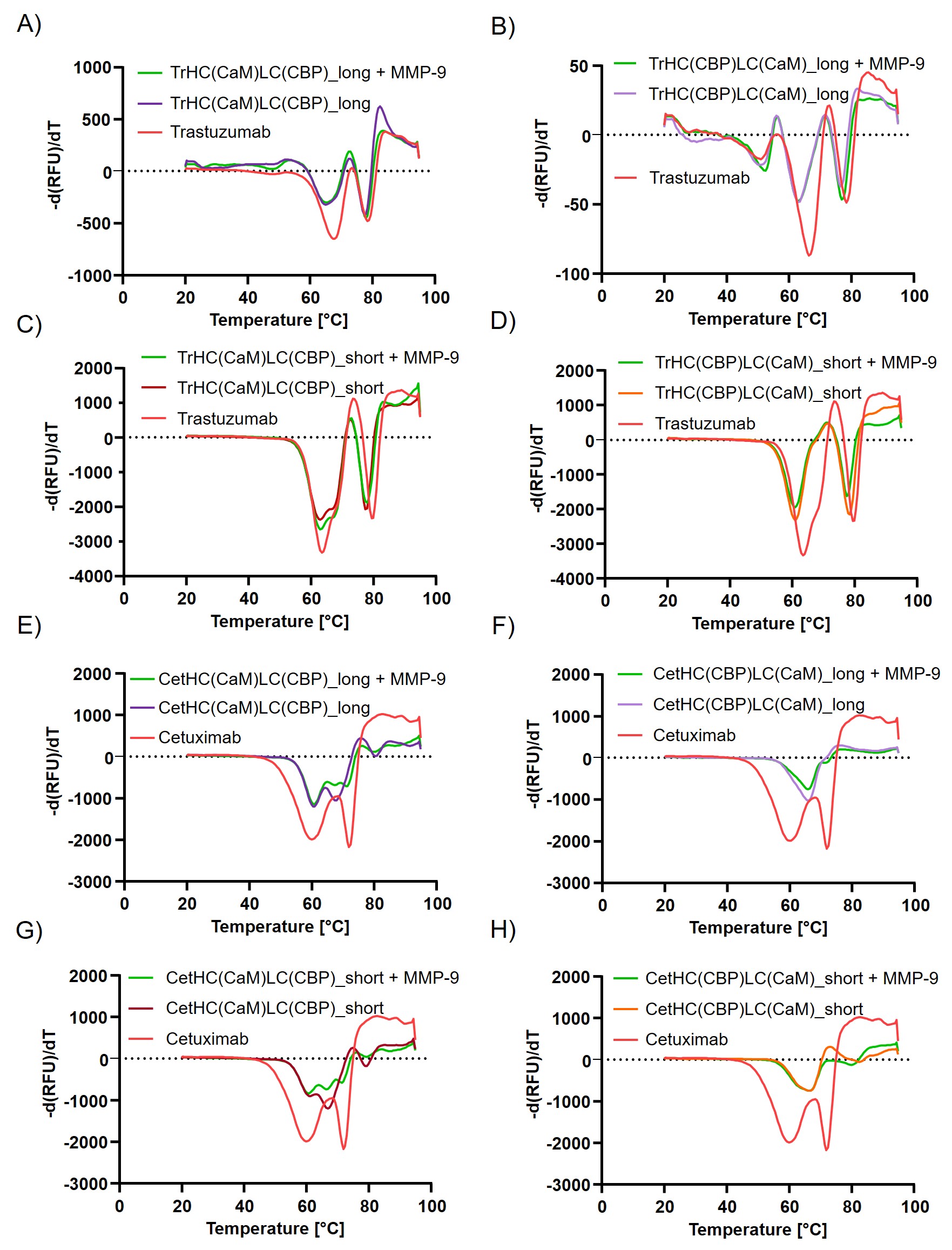
**

**Supplementary Figure 4. Thermal stability analysis of peptide clamp masked antibodies**. The temperature is shown on the x-axis, and derivative fluorescence decrease is shown on the y-axis. The unmodified trastuzumab or cetuximab is displayed in red, while the antibody masked with the peptide clamp is displayed in individual colors for each masked construct, and after MMP-9‑cleavage is displayed in green. **(A)** Melting point analysis of the trastuzumab construct with CaM fused to the V_H_ and CBP fused to V_L_ *via* the long MMP-9‑cleavable linker. **(B)** Melting point analysis of the trastuzumab construct with CBP fused to the V_H_ and CaM fused to V_L_ *via* the long MMP-9‑cleavable linker. **(C)** Melting point analysis of the trastuzumab construct with CaM fused to the V_H_ and CBP fused to V_L_ *via* the short MMP‑9‑cleavable linker. **(D)** Melting point analysis of the trastuzumab construct with CBP fused to the V_H_ and CaM fused to V_L_ *via* the short MMP-9‑cleavable linker. **(E)** Melting point analysis of the cetuximab construct with CaM fused to the V_H_ and CBP fused to V_L_ *via* the long MMP‑9‑cleavable linker. **(F)**Melting point analysis of the cetuximab construct with CBP fused to the V_H_ and CaM fused to V_L_ *via* the long MMP-9-cleavable linker. **(G)** Melting point analysis of the cetuximab construct with CaM fused to the V_H_ and CBP fused to V_L_ *via* the short MMP-9-cleavable linker. **(H)** Melting point analysis of the cetuximab construct with CBP fused to the V_H_ and CaM fused to V_L_ *via* the short MMP-9-cleavable linker.

**
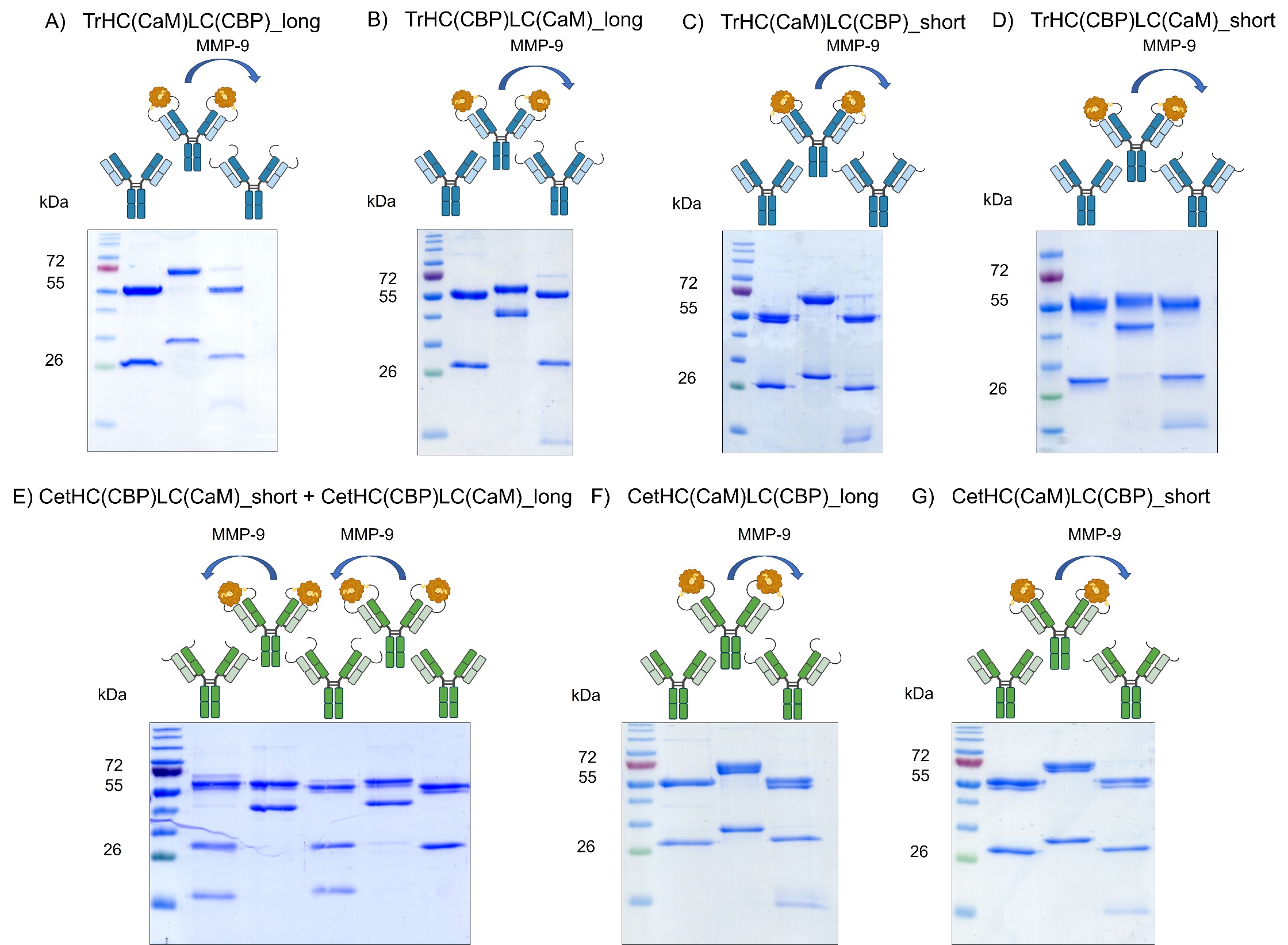
**

**Supplementary Figure 5. Analysis of peptide clamp linker hydrolysis by MMP-9 using reducing SDS-PAGE.** Panels A-D depict MMP-9-mediated linker hydrolysis of trastuzumab (molecular weights: HC approx. 50 kDa; LC approx. 23 kDa) masked by CaM (molecular weight: approx.17 kDa) and CBP fusion constructs. Demasking of cetuximab (molecular weights: HC approx. 50 kDa; LC approx. 23 kDa) fusion constructs masked by fusion of CaM and CBP are shown in Panels E‑G. **(A)** Linker hydrolysis of trastuzumab with CaM fused to the V_H_ domain and CBP fused to the V_L_ domain *via* the long MMP‑9-cleavable linker. **(B) ** Linker hydrolysis of trastuzumab with CBP fused to the V_H_ domain and CaM fused to the V_L_ domain *via* the long MMP-9-cleavable linker. **(C)**Linker hydrolysis of trastuzumab with CaM fused to the V_H_ domain and CBP fused to the V_L_ domain *via* the short MMP‑9‑cleavable linker. **(D)** Linker hydrolysis of trastuzumab with CBP fused to the V_H_ domain and CaM fused to the V_L_ domain *via* the short MMP-9-cleavable linker. **(E)**Demasking of cetuximab with CBP fused to the V_H_ and Calmodulin fused to the V_L_ domain *via* either the short (left) or long (right) MMP-9-cleavable linker. **(F)** Demasking ofcetuximab with CaM fused to the V_H_ domain and CBP fused to the V_L_ domain via the long MMP-9-cleavable linker. **(G)**Demasking of cetuximab with CaM fused to the V_H_, and CBP fused to the V_L_ *via* the short MMP-9 cleavable linker. For protein size estimation, the Color Prestained Protein Standard Broad Range (10–250 kDa, New England Biolabs) was used as a molecular weight reference. Created with Biorender.com.

**
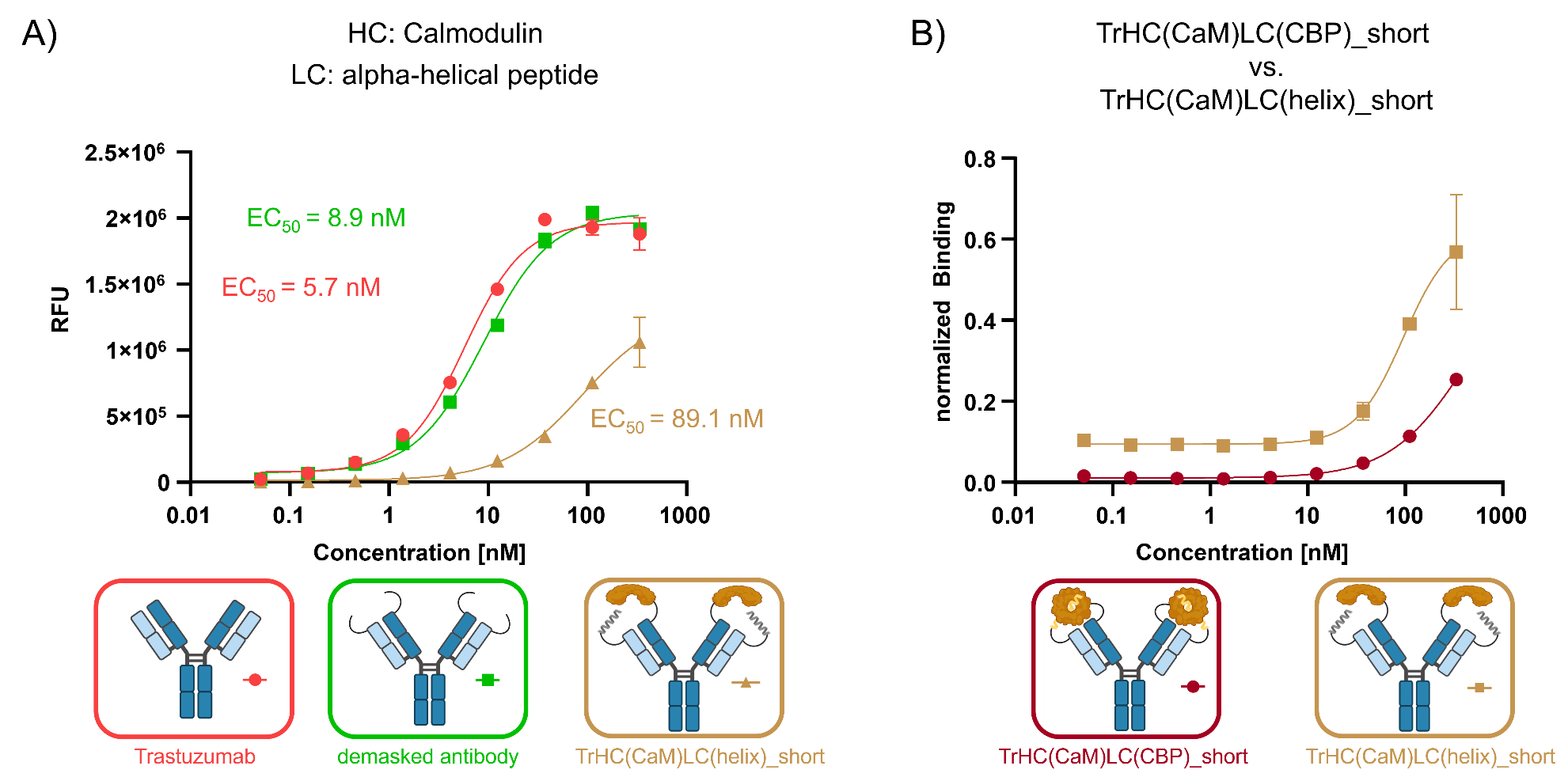
**

**Supplementary Figure 6. Evaluation of on-cell binding of trastuzumab masked with a Calmodulin and an alpha-helical peptide. (A)** Analysis of concentration-dependent on-cell binding on HER2 positive SK-BR-3 cells using flow cytometry. Antibody concentration (nM) is plotted on the x axis, and fluorescence intensity, measured in relative fluorescence units (RFU), is displayed on the y-axis. Trastuzumab was masked using CaM connected to the heavy chain *via* the short MMP-9 cleavable linker and a 13 amino acid alpha-helical peptide connected to the light chain using the long MMP-9 cleavable linker. Antibody binding was visualized using a PE-conjugated anti-human IgG detection antibody. Experiments were repeated twice, showing similar results. **(B)** Comparison of concentration-dependent binding of TrHC(CaM)LC(CBP)_short and TrHC(CaM)LC(helix)_short to HER2-positive SK-BR-3 cells. Antibody concentrations (nM) are shown on the x-axis, and normalized binding, calculated as RFU values of the constructs relative to trastuzumab, is shown on the y-axis.The figure displays binding of TrHC(CaM)LC(CBP)_short shown in Figure 2B, compared to binding of TrHC(CaM)LC(helix)_short shown in Figure S7A. Binding curves were fitted using a sigmoidal four-parameter logistic regression model. Error bars indicate the standard deviation of experimental triplicates. Created with Biorender.com


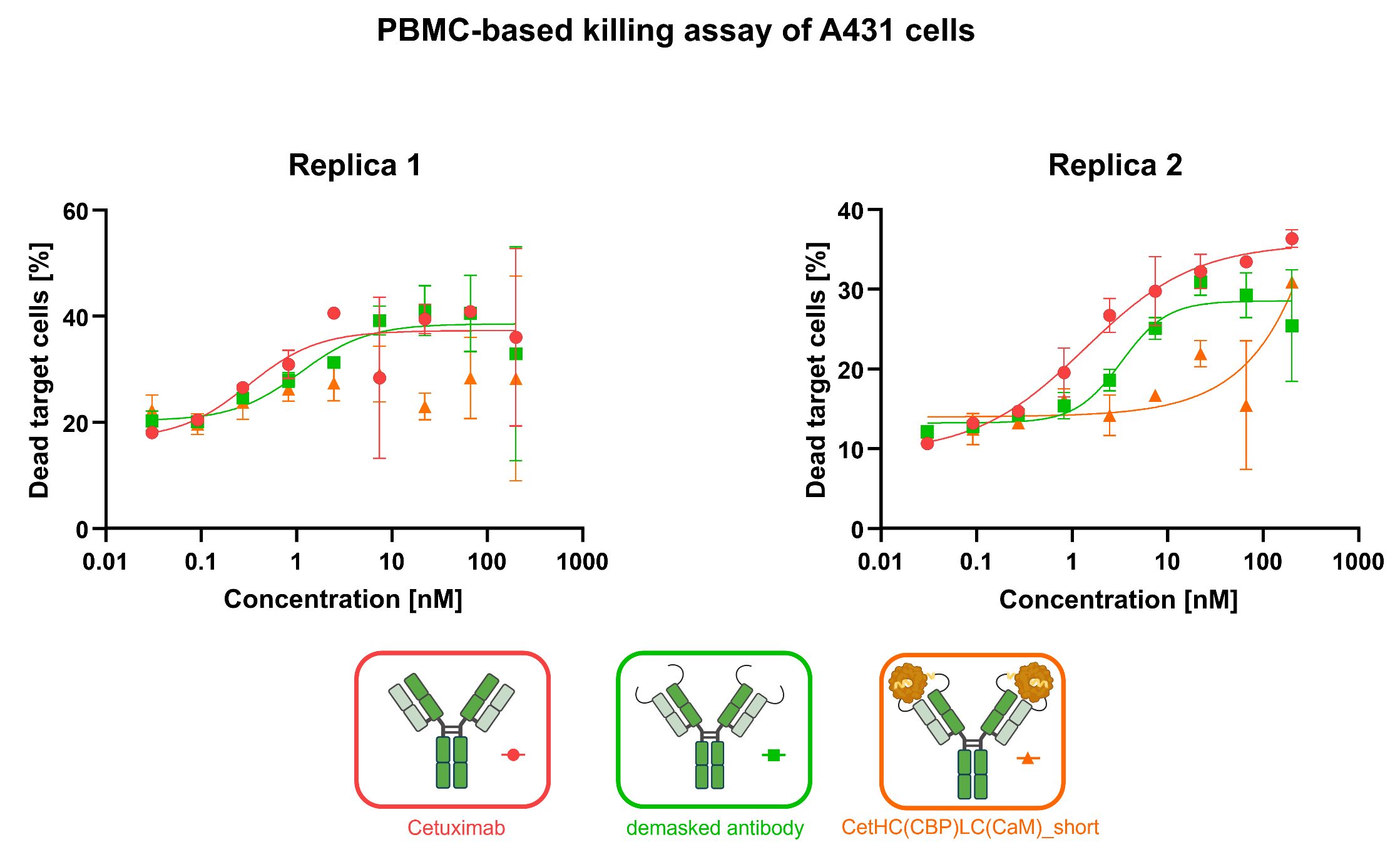


**Supplementary Figure 7. PBMC-based killing assay of A431 cells using Cetuximab and CetHC(CBP)LC(CaM)_short.** Analysis of ADCC-mediated PBMC-based killing of EGFR-overexpressing A431 cells by Cetuximab, as well as CetHC(CBP)LC(CaM) before and after MMP-9 mediated unmasking. Antibody concentration is plotted on the x-axis and the percentage of dead target cells is plotted on the y-axis. Cetuximab was masked fusing CBP to the heavy chain and CaM to the light chain utilizing the short MMP-9 cleavable linker. Curves were fitted using a sigmoidal four-parameter logistic regression model. Error bars indicate the standard deviation of experimental duplicates. Experiments were repeated twice shown on the right and left.


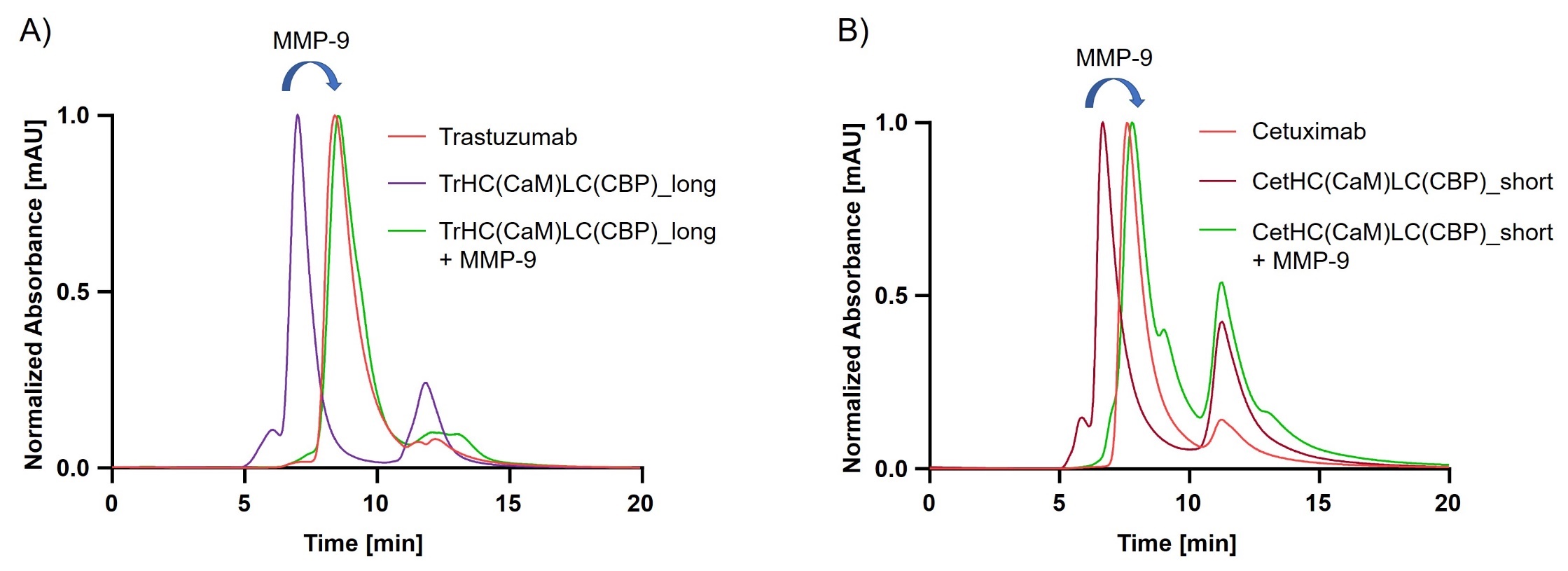


**Supplementary Figure 8. Analytical SEC of the peptide clamp masked trastuzumab and cetuximab before and after MMP-9 linker cleavage.** The time in minutes is shown on the x-axis, and the normalized absorbance (mAU) is shown on the y-axis. **(A)** Analytical SEC of TrHC(CaM)LC(CBP)_long before and after MMP-9 cleavage. The unmodified trastuzumab is shown in red, while the peptide clamp masked antibody TrHC(CaM)LC(CBP)_long is shown in purple. The masked antibody after MMP-9 cleavage is shown in green. **(B)** Analytical SEC of CetHC(CaM)LC(CBP)_short before and after MMP-9 cleavage. The unmodified trastuzumab is shown in red, while the peptide clamp masked antibody CetHC(CaM)LC(CBP)_short is shown in dark red. The masked antibody after MMP-9 cleavage is shown in green.


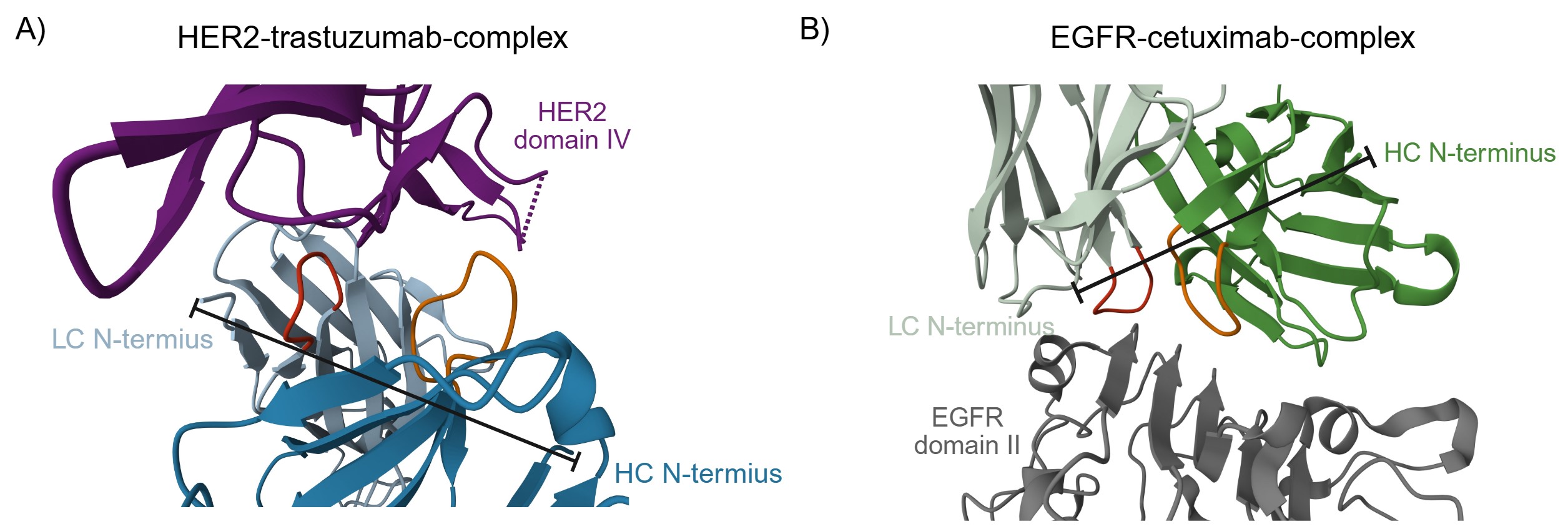


**Supplementary Figure 9. Trastuzumab Fab-HER2-complex and cetuximab Fab-EGFR complex. (A)** The trastuzumab heavy chain is shown in dark blue, and the light chain is shown in light blue, while the HER2 domain IV is shown in purple. The heavy chain CDR3 is colored orange, and the light chain CDR3 is colored red. A black line indicates the distance between the N-termini of the heavy and light chains. The light chain N-terminus is closer to both CDR3s of heavy and light chain than the heavy chain N-terminus. Structure adapted from PDB: 1N8Z. **(B)** The cetuximab heavy chain is shown in dark green, and the light chain is shown in light green, while the HER2 domain IV is shown in grey. The heavy chain CDR3 is colored orange, and the light chain CDR3 is colored red. A black line indicates the distance between the N-termini of the heavy and light chains. The light chain N-terminus is closer to both CDR3s of heavy and light chain than the heavy chain N-terminus. Structure adapted from PDB: 1YY9.
